# Supplementary material for: Accounting for environmental and fishery management factors when standardizing CPUE data from a scientific survey: A case study for Nephrops norvegicus in the Pomo Pits area (Central Adriatic Sea)
Source: PLoS One. 2022 Jul 14;17(7):e0270703. doi: 10.1371/journal.pone.0270703 (PMC9282463; doi:10.1371/journal.pone.0270703)
Supplement: S1 File — S1-S5 Figs showing maps about the management measures implemented within the study area since 2015, bubble plots by year of normalized biomass and density CPUE for each haul of both spring and autumn surveys. S6-S11 Figs showing environmental maps of bottom salinity, bottom dissolved oxygen and bottom temperature by year for both spring and autumn time series. S12-S17 Figs showing plot about the residual analysis for the final model for both biomass and density indices together with prediction maps of the related standard errors for both time series. (DOCX) [file pone.0270703.s001.docx]

**Accounting for environmental and fishery management factors when standardizing CPUE data from a scientific survey: A**  **case study for *Nephrops norvegicus* in the Pomo Pits area (Central Adriatic Sea)**

Chiarini M.^1,2,^*, Guicciardi S.^1^, Angelini S.^1,3^, Tuck I.D.^4^, Grilli F.^1^, Penna P.^1^, Domenichetti F.^1^, Canduci G.^1^,Belardinelli A.^1^, Santojanni A.^1^, Milone N.^5^, Arneri E.^1,5^, Medvešek D.^6^, Isajlovic I.^6^, Vrgoč N.^6^, Martinelli M.^1^

^1^National Research Council – Institute of Marine Biological Resources and Biotechnologies (CNR IRBIM), Ancona (Italy)

^2^Department of Biological, Geological and Environmental Sciences, University of Bologna (UNIBO), Bologna (Italy)

^3^ Fano Marine Center, The Inter-Institute Center for Research on Marine Biodiversity, Resources and Biotechnologies, Fano (Italy)

^4^National Institute of Water and Atmosphere (NIWA), Auckland (New Zealand)

^5^Food and Agriculture Organization (FAO), Rome (Italy)

^6^Institute of Oceanography and Fisheries (IOF), Split (Croatia)

*Corresponding author: e-mail: [matteo.chiarini5@unibo.it](mailto:matteo.chiarini5@unibo.it) (MC)

ORCID iD: <https://orcid.org/0000-0002-6086-3372>

**Supporting information**

**S1 Table.** **Parametric one-way ANOVA for abundance models (kg/km^2^).** Analysis of the variance of AIC values across the four tested models for abundance. Hence, “model” is the factor, “Df” is the number of degree of freedom, “Sum Sq” represent the sums of squares (i.e. the distances of each point to the mean), “Mean Sq” is the mean of squares, “F” is the ratio of the sum of squares, “Pr(>F)” is the combination of F-statistic with the degrees of freedom. Asterisk (*) refers to the level of significance (>0.05).

|  | Df | Sum Sq | Mean Sq | F | Pr(>F) |
| --- | --- | --- | --- | --- | --- |
| model | 3 | 105151 | 35050 | 501.7 | <2e-16 * |
| Residuals | 396 | 27664 | 70 |  |  |

**S2 Table.** **Parametric one-way ANOVA for density models (N/km^2^).** Analysis of the variance of AIC values across the four tested models for density. Hence, “model” is the factor “Df” is the number of degree of freedom, “Sum Sq” represent the sums of squares (i.e. the distances of each point to the mean), “Mean Sq” is the mean of squares, “F” is the ratio of the sum of squares, “Pr(>F)” is the combination of F-statistic with the degrees of freedom. Asterisk (*) refers to the level of significance (>0.05).

|  | Df | Sum Sq | Mean Sq | F | Pr(>F) |
| --- | --- | --- | --- | --- | --- |
| model | 3 | 82820 | 27607 | 176.1 | <2e-16 * |
| Residuals | 396 | 62094 | 157 |  |  |

**S3 Table.** **Tukey multiple comparisons among AIC values of “modINITIAL”, “modNOEM”, “modNOM” and “modNOE” for abundance models (kg/km^2^).** Tukey post-hoc test (95% family-wise confidence level) conducted on averaged AIC values calculated from the 10 k-fold cross validation repeated 10 times for each combination of the four tested models (“modINITIAL”, “modNOEM”, “modNOE”, “modNOM”) for biomass index. “diff” indicates the difference between values, “lwr” is the lower bound of the confidence interval while “upr” is the upper bound, and “p adj” is the adjusted p-value.

|  | diff | lwr | upr | P adj |
| --- | --- | --- | --- | --- |
| modNOEM - modINITIAL | -37.147301 | -40.1968950 | -34.097707 | 0.0000000 |
| modNOEM - modNOM | -6.463387 | -9.5129805 | -3.413793 | 0.0000005 |
| modNOEM - modNOE | -33.290466 | -36.3400595 | -30.240872 | 0.0000000 |
| modNOM - modINITIAL | 30.683914 | 27.6343206 | 33.733508 | 0.0000000 |
| modNOE - modINITIAL | 3.856835 | 0.8072416 | 6.906429 | 0.0065551 |
| modNOE - modNOM | -26.827079 | -29.8766729 | -23.777485 | 0.0000000 |

**S4 Table.** **Tukey multiple comparisons among AIC values of “modINITIAL”, “modNOEM”, “modNOM” and “modNOE” for density models (N/km^2^).** Tukey post-hoc test (95% family-wise confidence level) conducted on averaged AIC values calculated from the 10 k-fold cross validation repeated 10 times for each combination of the four tested models (“modINITIAL”, “modNOEM”,“modNOE”, “modNOM”) for density index. “diff” indicates the difference between values, “lwr” is the lower bound of the confidence interval while “upr” is the upper bound, and “p adj” is the adjusted p-value.

|  | diff | lwr | upr | p adj |
| --- | --- | --- | --- | --- |
| modNOEM - modINITIAL | -36.925820 | -41.494659 | -32.35698 | 0.00e+00 |
| modNOEM - modNOM | -7.769049 | -12.337888 | -3.20021 | 8.67e-05 |
| modNOEM - modNOE | -24.573393 | -29.142232 | -20.00455 | 0.00e+00 |
| modNOM - modINITIAL | 29.156771 | 24.587932 | 33.72561 | 0.0000000 |
| modNOE - modINITIAL | 12.352427 | 7.783588 | 16.92127 | 0.0000000 |
| modNOE - modNOM | -16.804344 | -21.373183 | -12.23551 | 0.0000000 |

**S5 Table.** **Mean AIC of “mod INITIAL”, “modNOEM”, “mod NOM” and “mod NOE” for both biomass (kg/km^2^) and density (N/km^2^) indices.** Mean AIC with standard deviation out of 100 AIC values from the first 10 k-fold cross validation repeated 10 times for each of the three four models (“modINITIAL”, “modNOEM”, “modNOE” and “modNOM”) for both biomass and density indices.

|  | Biomass (kg/km2) | Density (N/km2) |
| --- | --- | --- |
| modINITIAL | 677.485 ± 8.055 | 1407.745 ± 12.051 |
| modNOEM | 714.633 ± 8.267 | 1444.671 ± 12.668 |
| modNOM | 708.169 ± 8.586 | 1436.902 ± 11.663 |
| modNOE | 681.342 ± 8.515 | 1420.097 ± 13.619 |

**S6 Table.** **Mean RMSE of “mod INITIAL” and “mod FINAL” for both biomass (kg/km^2^) and density (N/km^2^) indices**. Mean RMSE with standard deviation out of 100 RMSE values from the second 10 k-fold cross validation repeated 10 times for the initial model (“modINITIAL”) and one including only significant terms (“modFINAL”) for both biomass and density indices.

|  | Biomass index (kg/km^2^) | Density index (N/km^2^) |
| --- | --- | --- |
| modINITIAL | 21.155 ± 7.29 | 1834.191 ± 935.640 |
| modFINAL | 20.727 ± 7.357 | 1755.832 ± 839.267 |

**S7 Table. Percentages of deviance explained by each term of “mod FINAL” for both biomass (kg/km^2^) and density (N/km^2^) indices.** Percentages (%) were calculated on the total explained deviance of each model according to Wood et al. [96]

| Covariate | Biomass index (kg/km^2^) | Density index (N/km^2^) |
| --- | --- | --- |
| Yr:Fishery | 33.4351145 | 34.83779972 |
| Y | 5.801526718 | 3.244005642 |
| D | 24.73282443 | 25.10578279 |
| Oxy | 6.870229008 | 8.74471086 |
| Sal | 7.938931298 | 10.1551481 |
| ToD | 10.38167939 | 7.757404795 |
| Fishery | 10.83969466 | 10.1551481 |


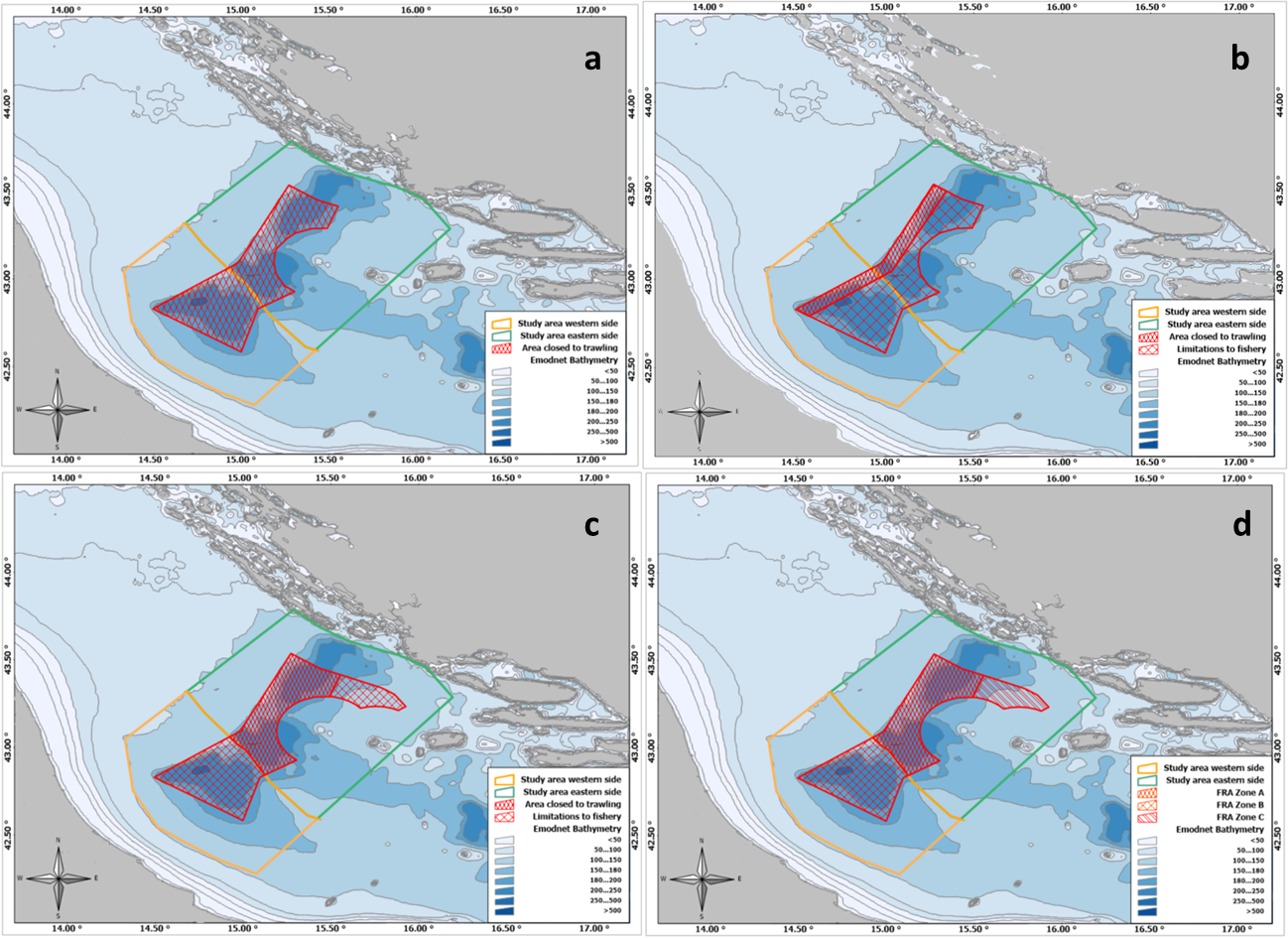


**S1 Fig. Management measures implemented within the study area since July 2015.** The maps show the bathymetry (source: [75]) and the boundaries of the study area, with western (orange polygon) and eastern (green polygon) sides divided by the Adriatic midline (source: [76]), and the spatial coverage of the management measures (inner red bounded polygons) implemented over the years in the Pomo/Jabuka Pits. Panel a shows (red dense grid) the extension of the first partial closure to trawling activities from 26/07/2015 to 16/10/2016 (Italian Ministerial Decree n.162 03/07/2015; Croatian Government Ordinance n.1533 20/07/2015). Panel b presents the area (red sparse grid) where a limited number of licences and fishing days for trawlers are allowed and the area (red dense grid) closed to all fishing activities from 01/10/2016 to 31/08/2017 (Italian Ministerial Decree n.17064 19/10/2016; Croatian Government Ordinance n.1106 17/05/2017). Panel c reports the area closed to trawling activities (red dense grid) from 01/09/2107 and the areas closed to all fishing activities (red sparse grids) until 31/10/2017 and then subject to limitations to fishery (Italian Ministerial Decree n.466 01/06/2017). Panel d refers to the Fishery Restricted Area implemented in the Pomo/Jabuka Pits by the GFCM/41/2017/3 Recommendation of October 2017, which establishes the fishery ban zone (A) and the two buffer zones subjected to limitations (B and C).


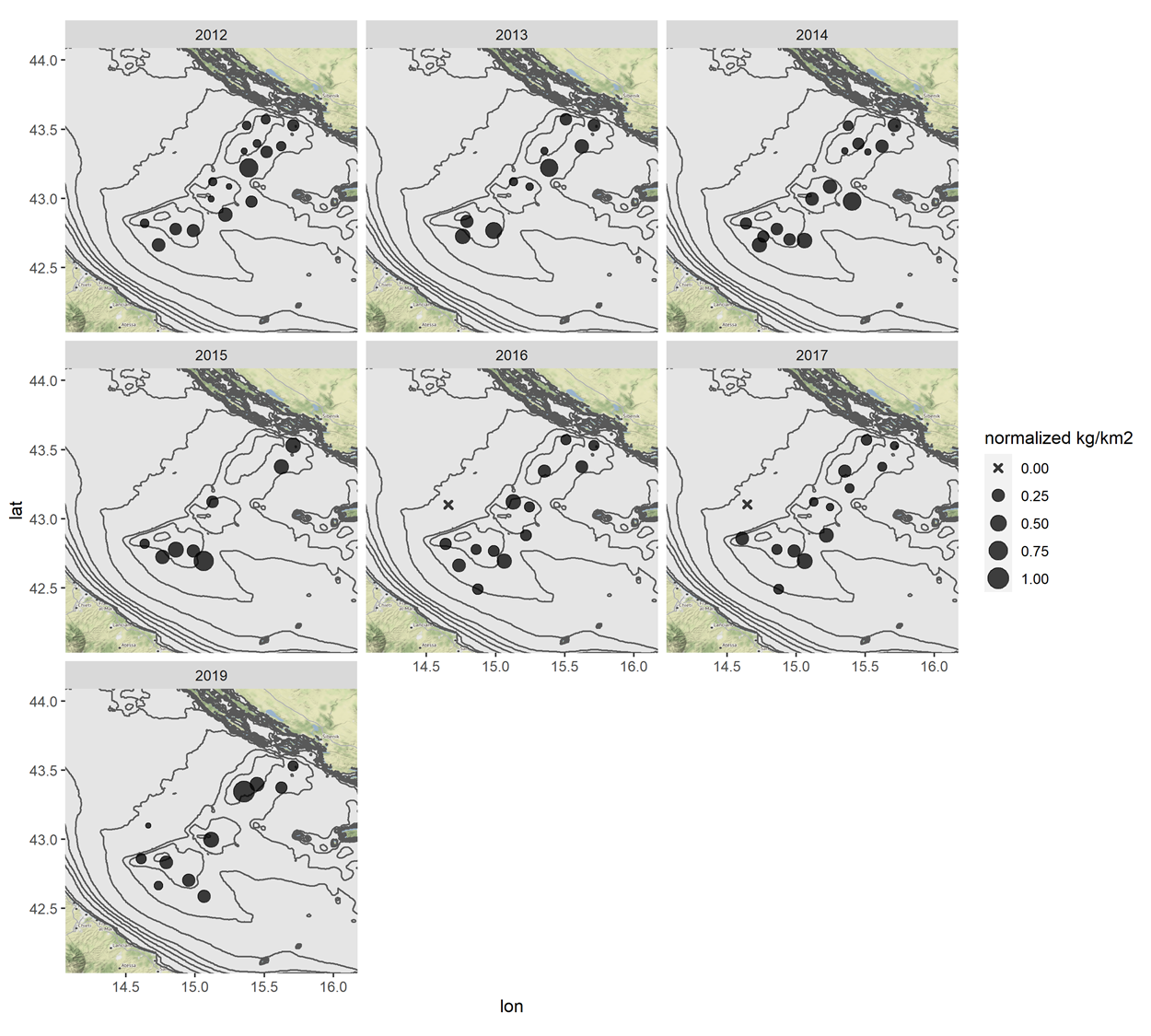
 **S2 Fig.** **Bubble Plots by year of normalized biomass CPUE calculated for each hauls of spring survey.**  Maps were made using the ggmap package [105] for R. Bathymetry layer source: [75]. Map tiles by Stamen Design, under CC BY Data by OpenStreetMap, under ODbL.


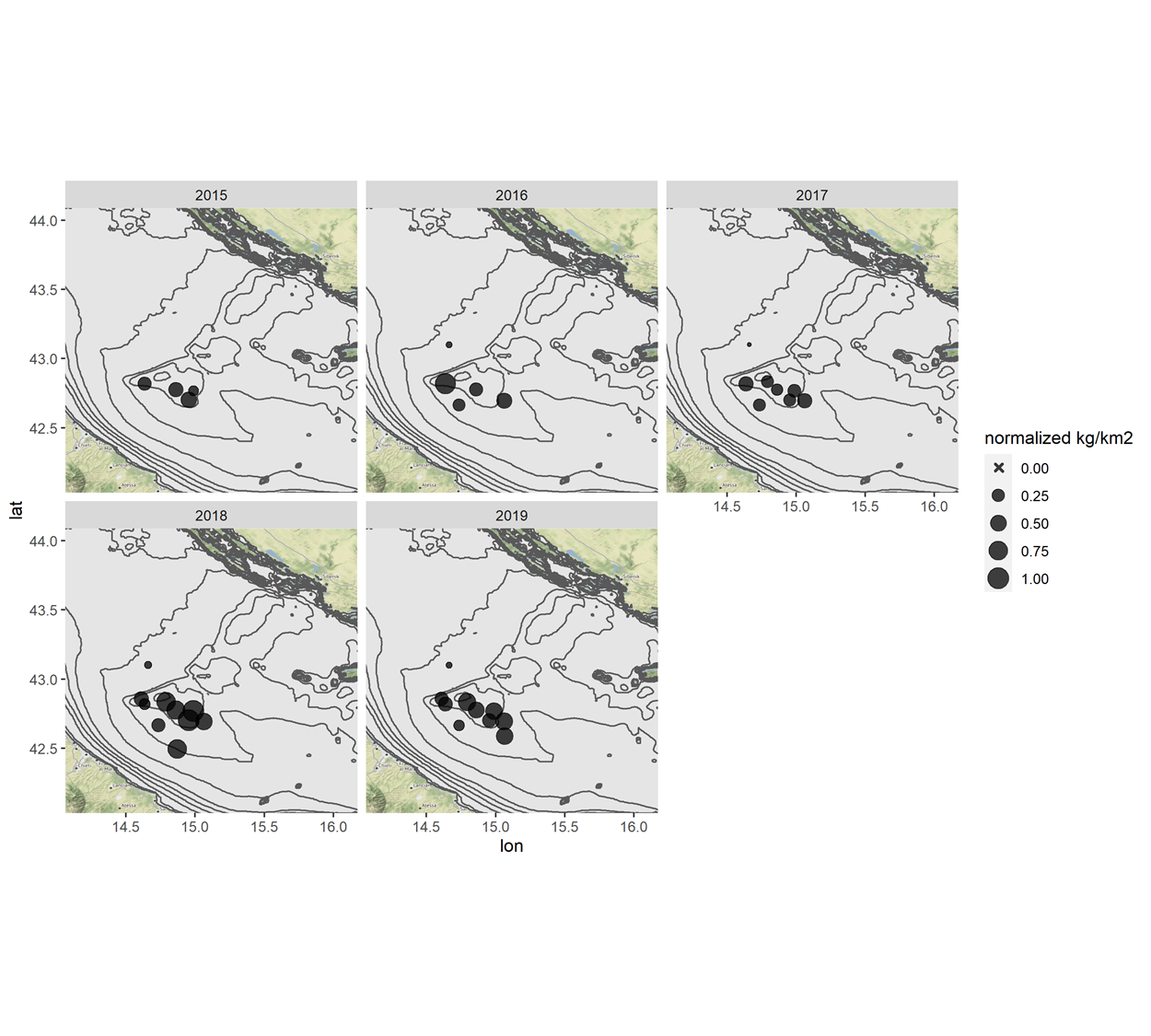
**S3 Fig.** **Bubble Plots by year of normalized biomass CPUE calculated for each hauls of autumn survey.** Maps were made using the ggmap package [105] for R. Bathymetry layer source: [75]. Map tiles by Stamen Design, under CC BY Data by OpenStreetMap, under ODbL.


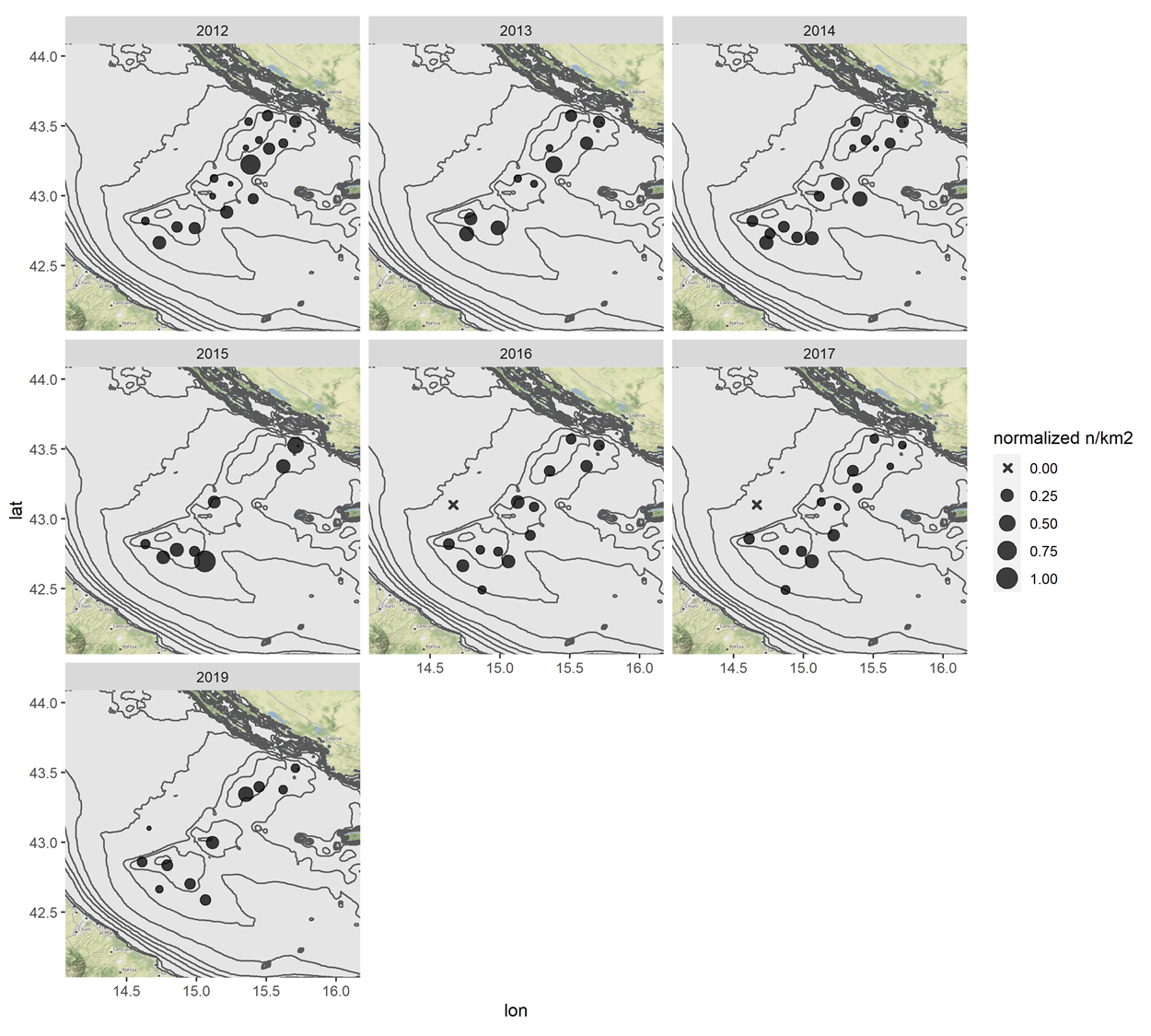
 **S4 Fig.** **Bubble Plots by year of normalized density CPUE calculated for each hauls of spring survey.**  Maps were made using the ggmap package [105] for R. Bathymetry layer source: [75]. Map tiles by Stamen Design, under CC BY Data by OpenStreetMap, under ODbL.


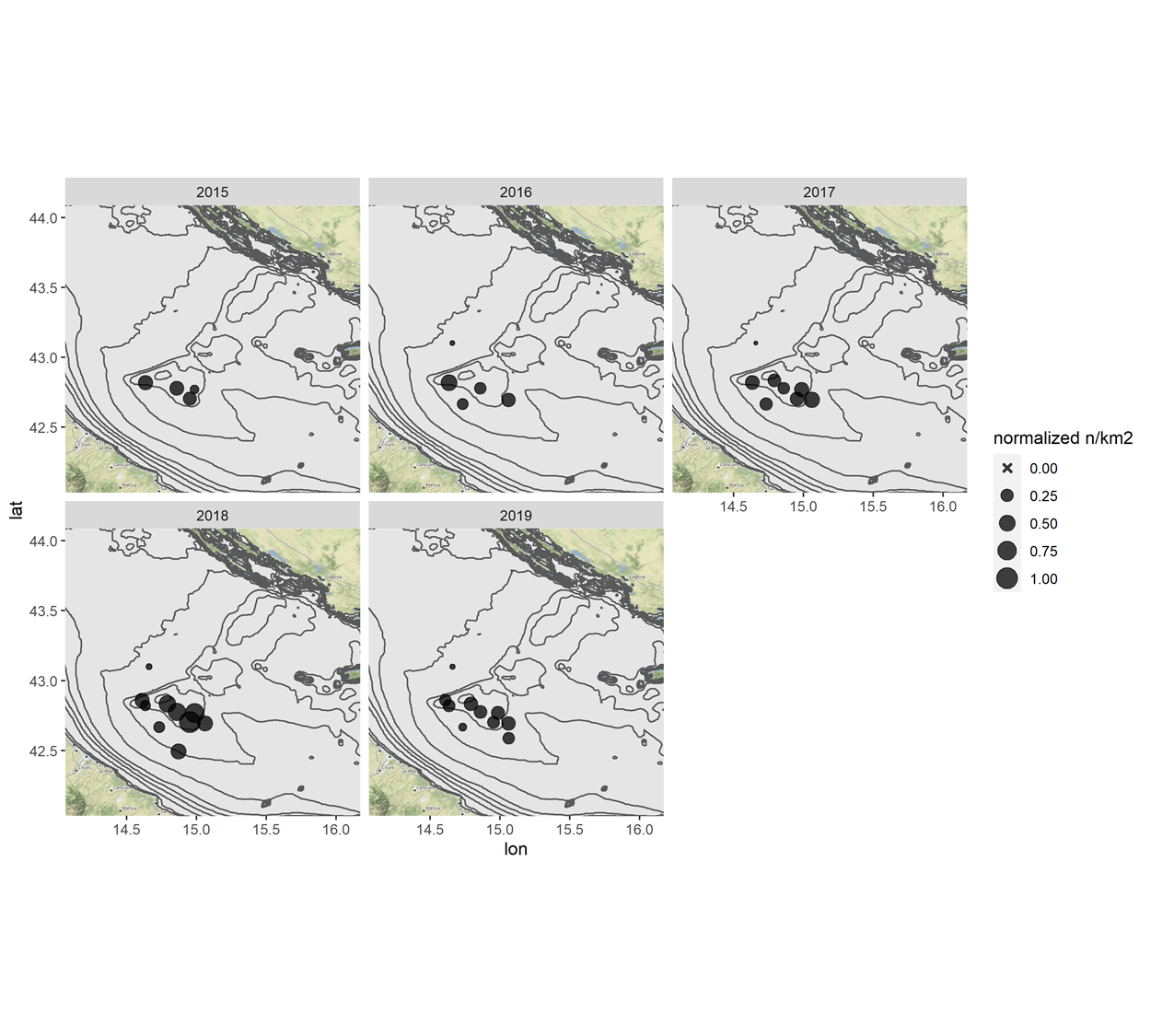
**S5 Fig.** **Bubble Plots by year of normalized density CPUE calculated for each hauls of autumn survey.** Maps were made using the ggmap package [105] for R. Bathymetry layer source: [75]. Map tiles by Stamen Design, under CC BY Data by OpenStreetMap, under ODbL.

**Environmental maps**

**S6 Fig.** **Maps of bottom salinity values for the spring time series.** Bathymetry layer source: [75].


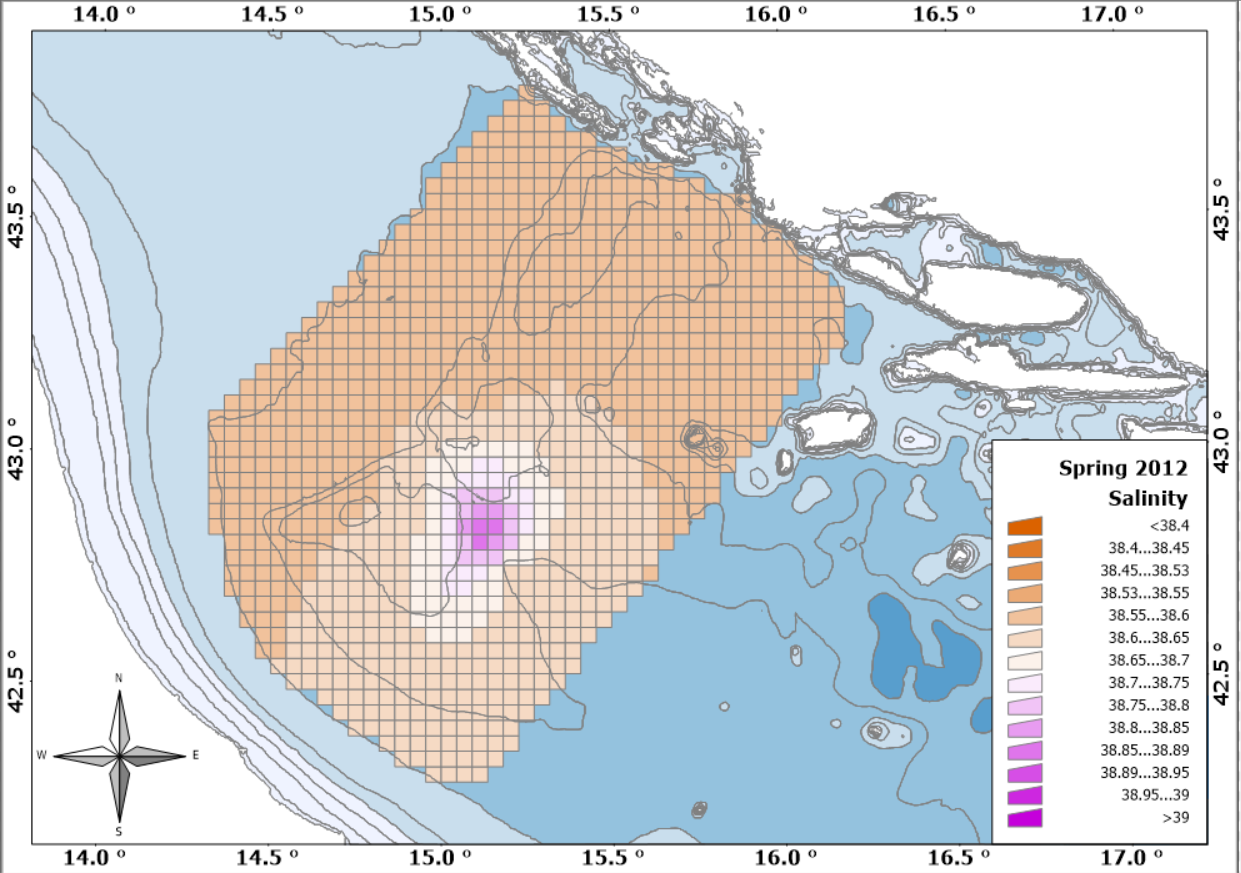


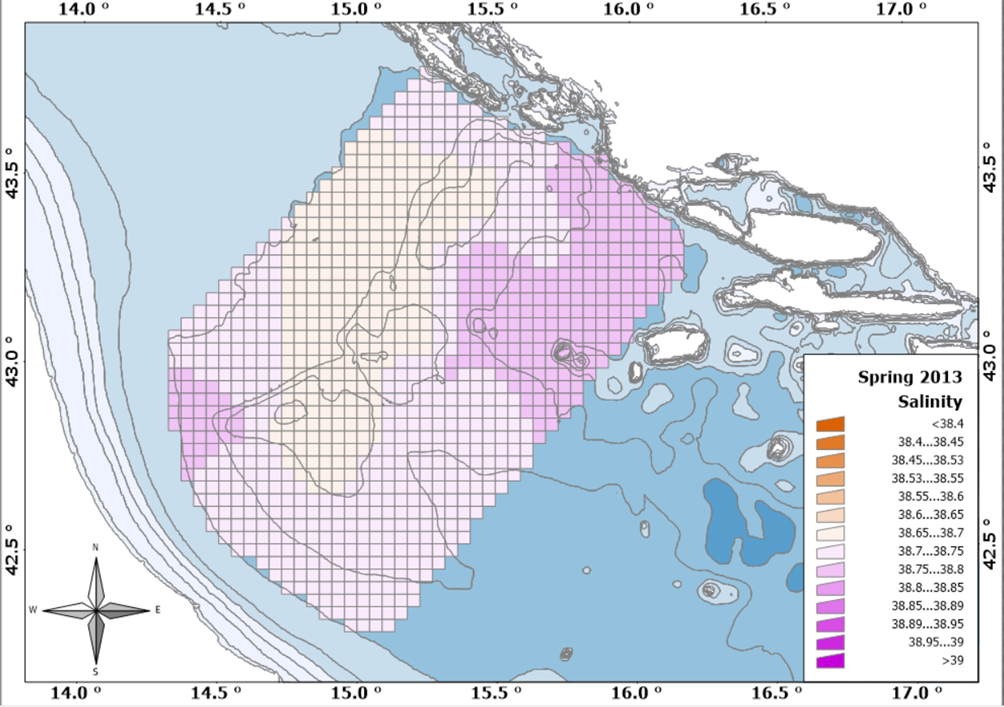


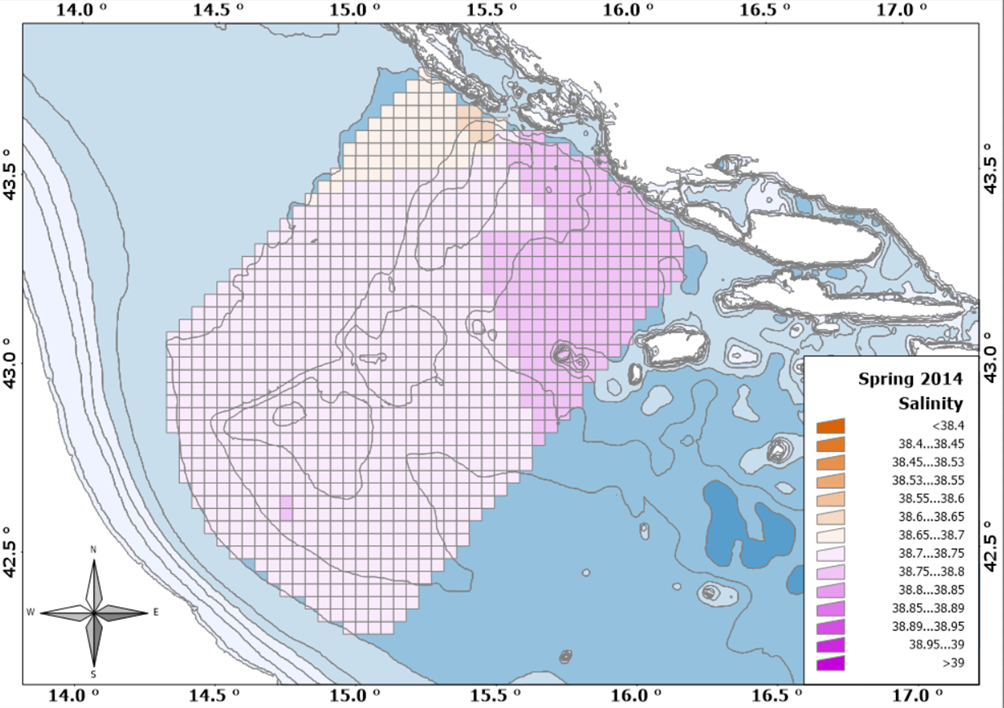


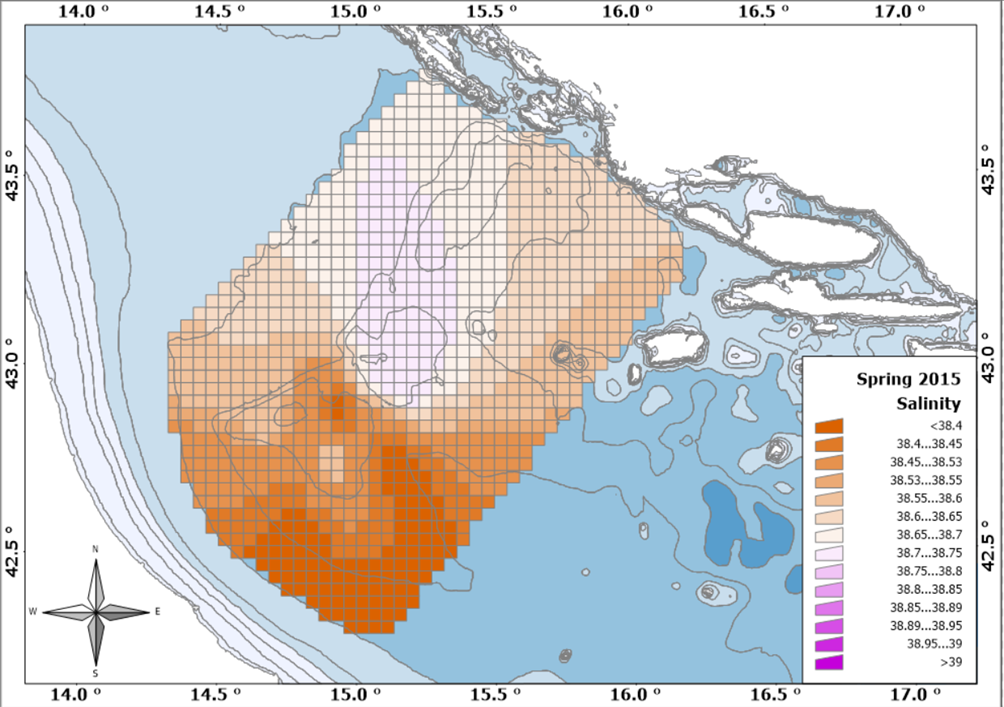


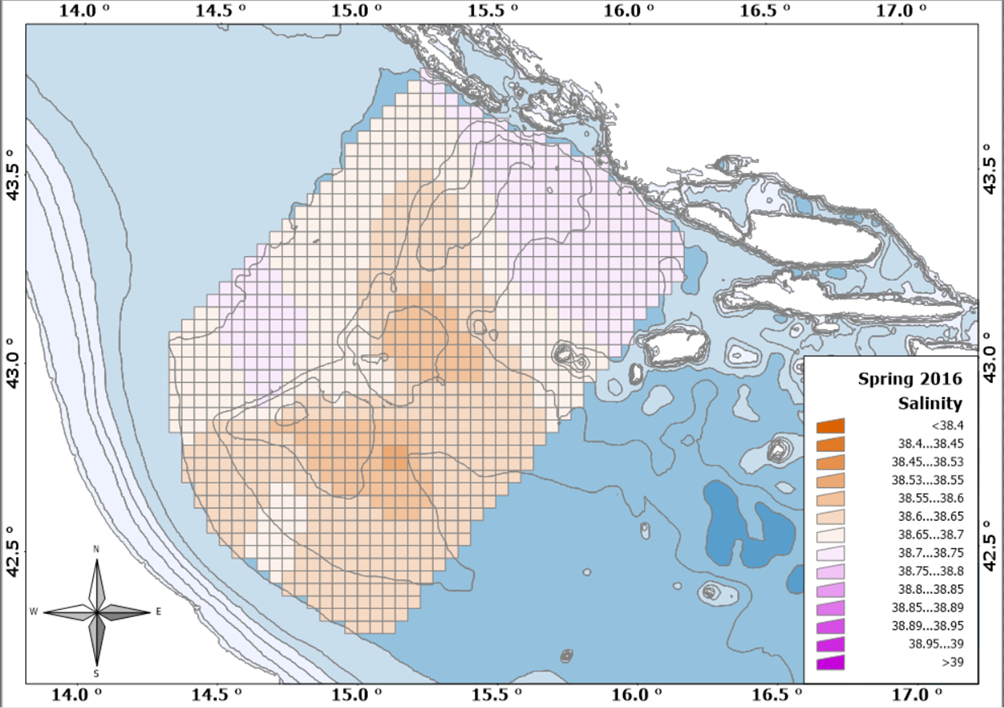


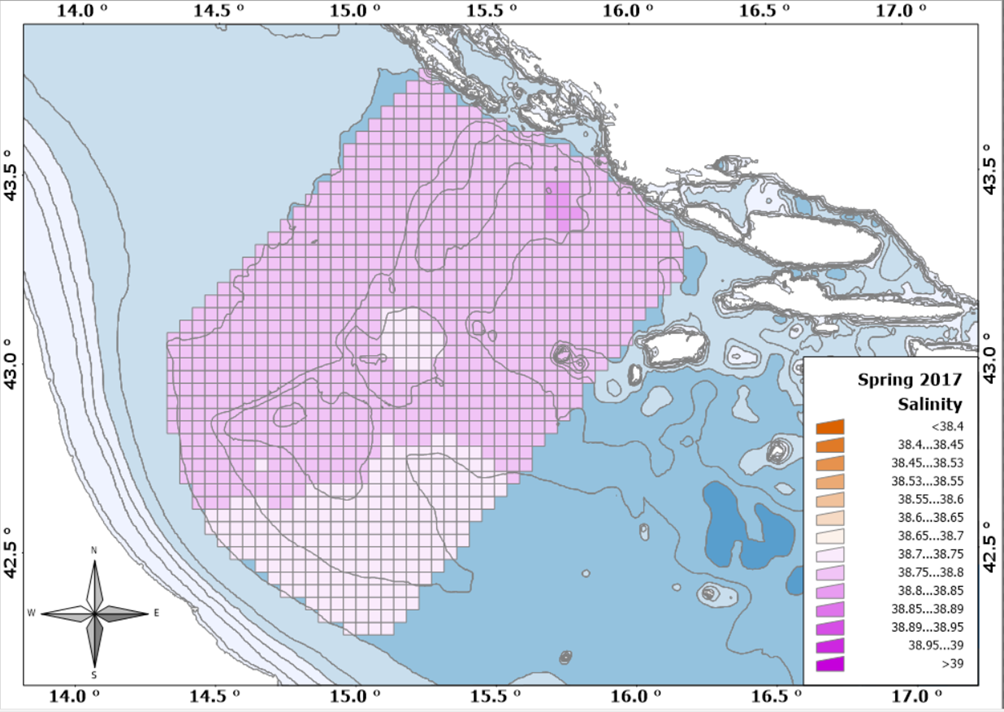


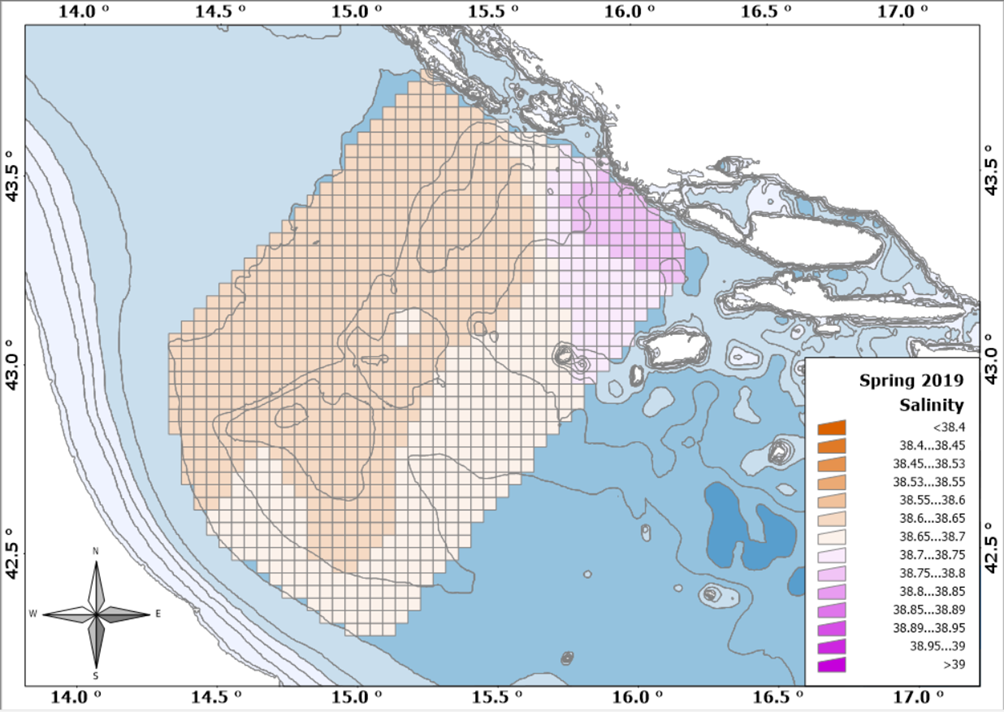


**S7 Fig. Maps of bottom salinity values for the autumn time series.** Bathymetry layer source: [75].


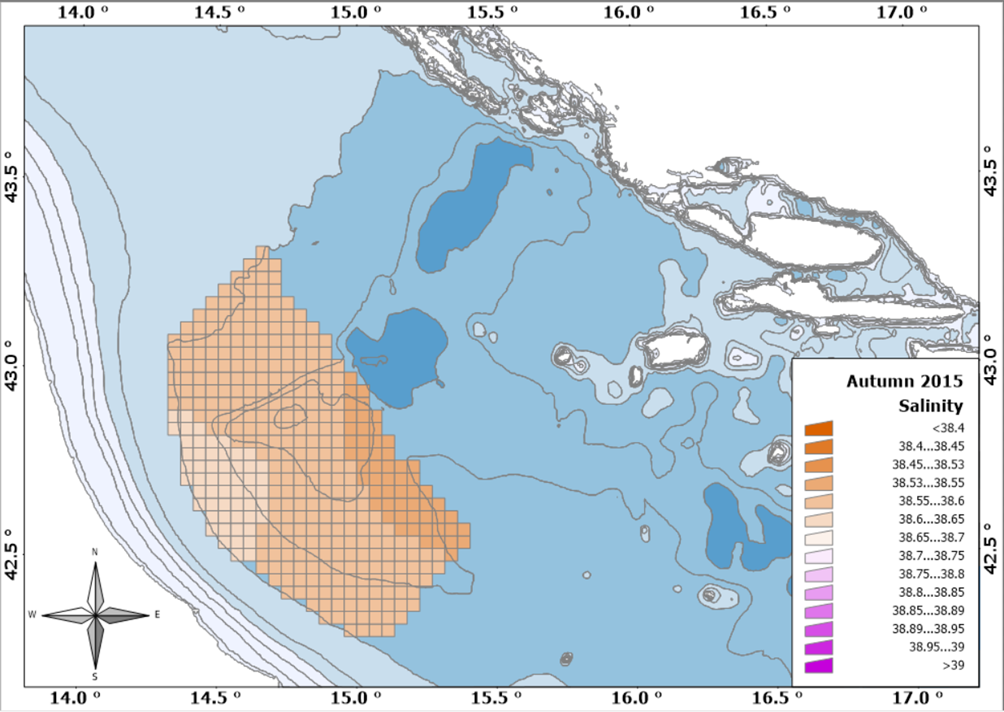


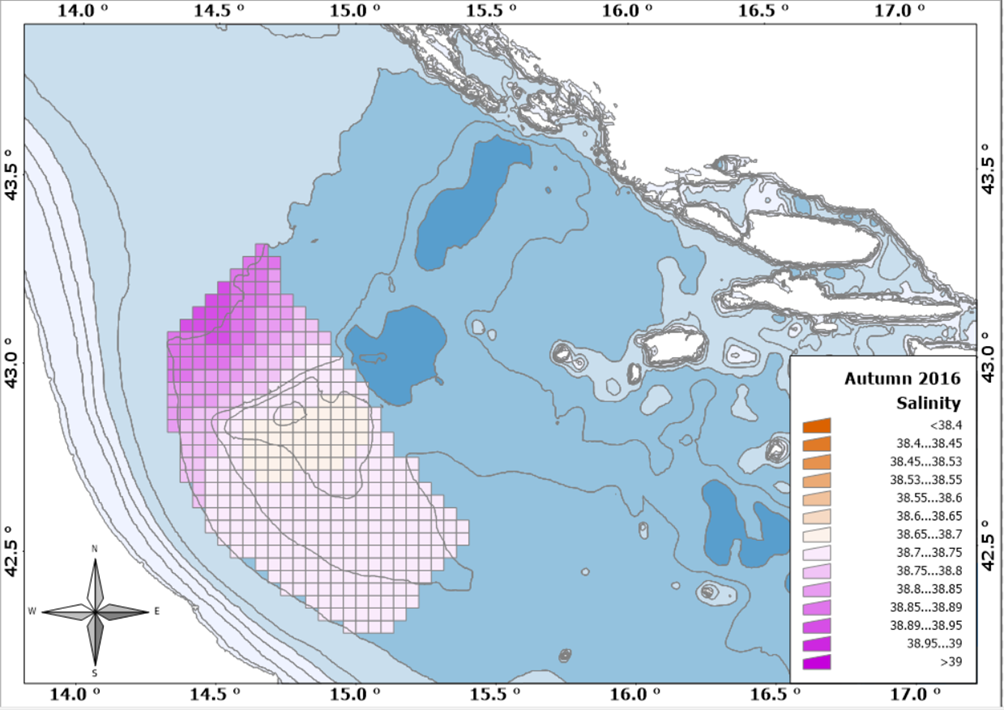


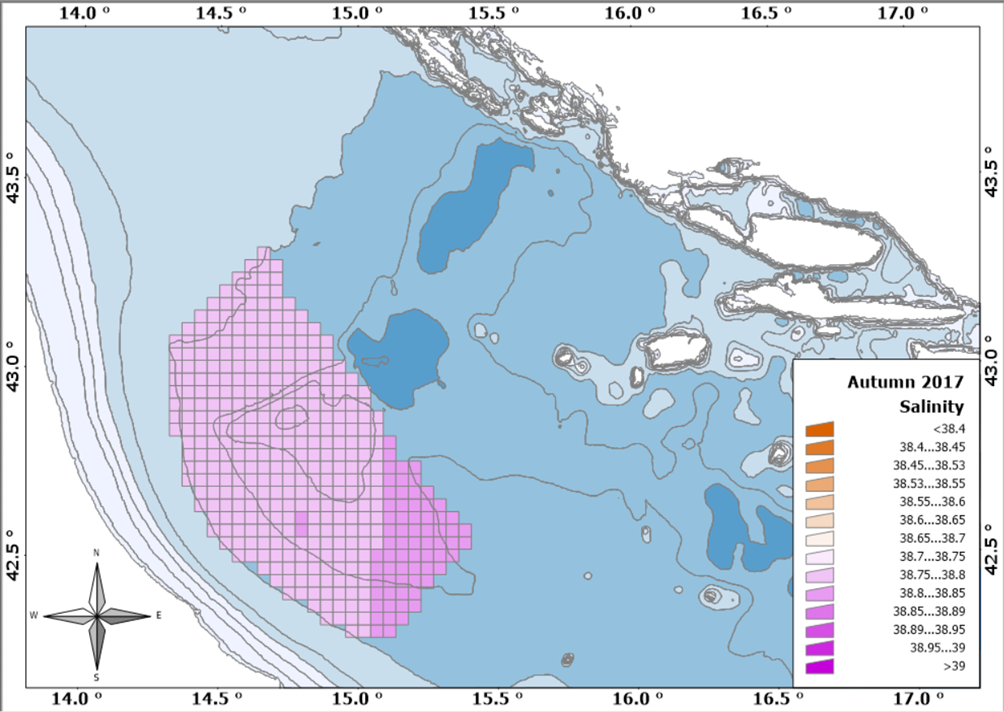


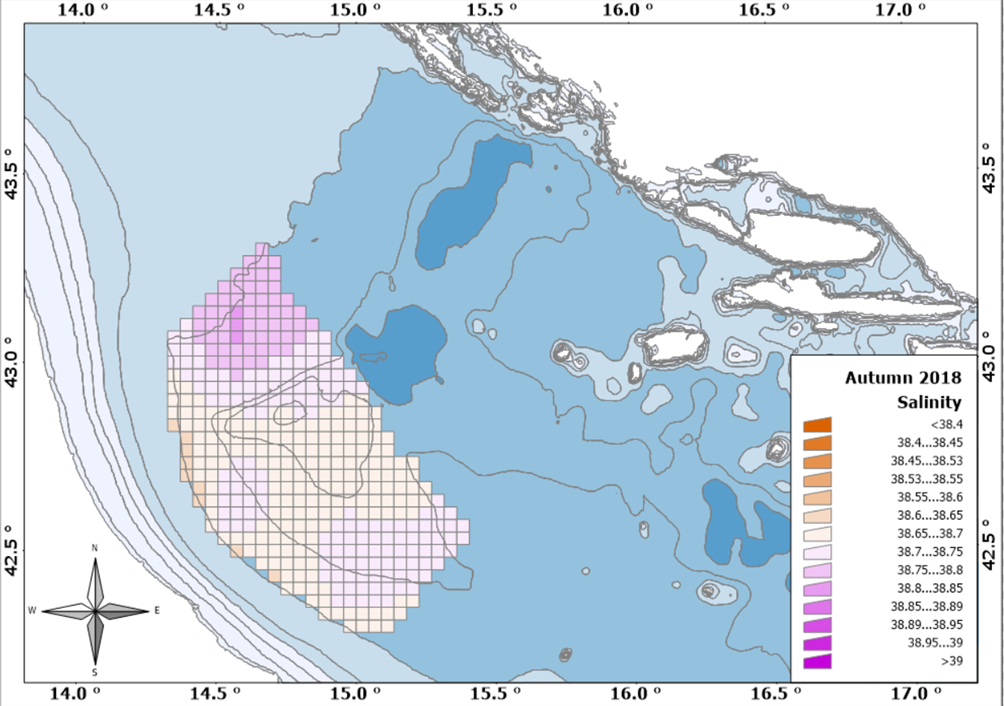


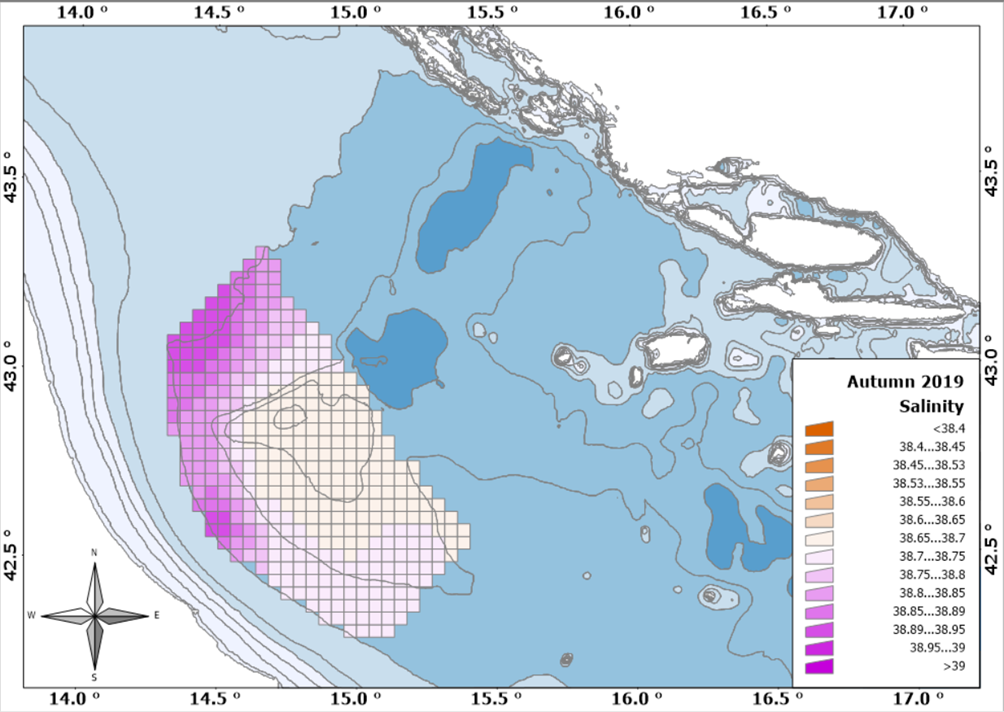


**S8 Fig.** **Maps of bottom dissolved oxygen saturation percentage for the spring time series.** Bathymetry layer source: [75].


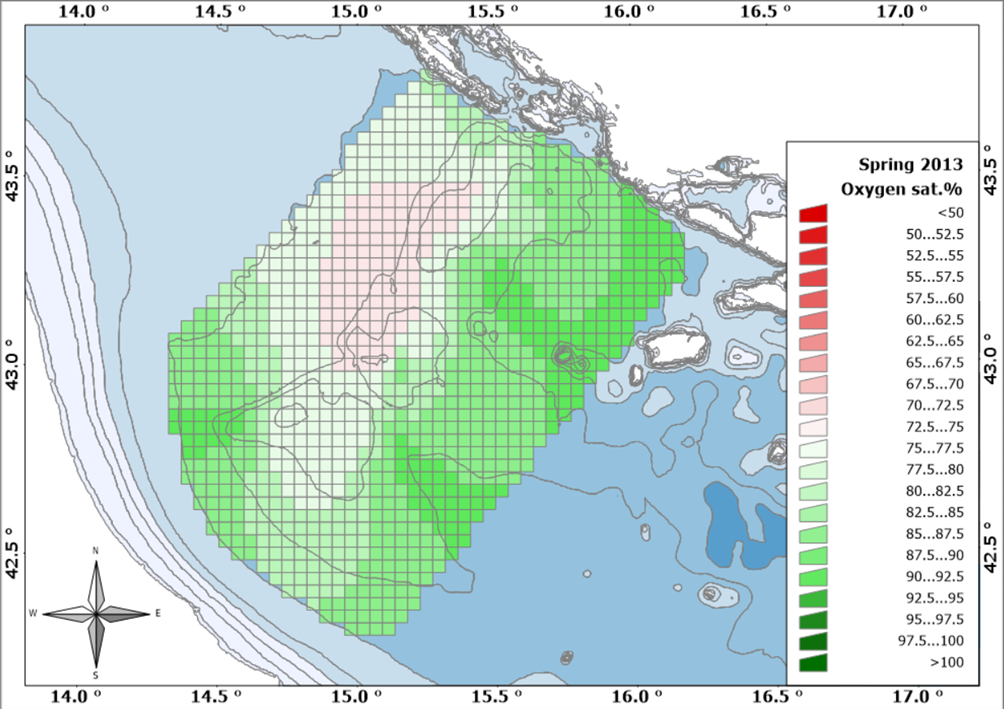


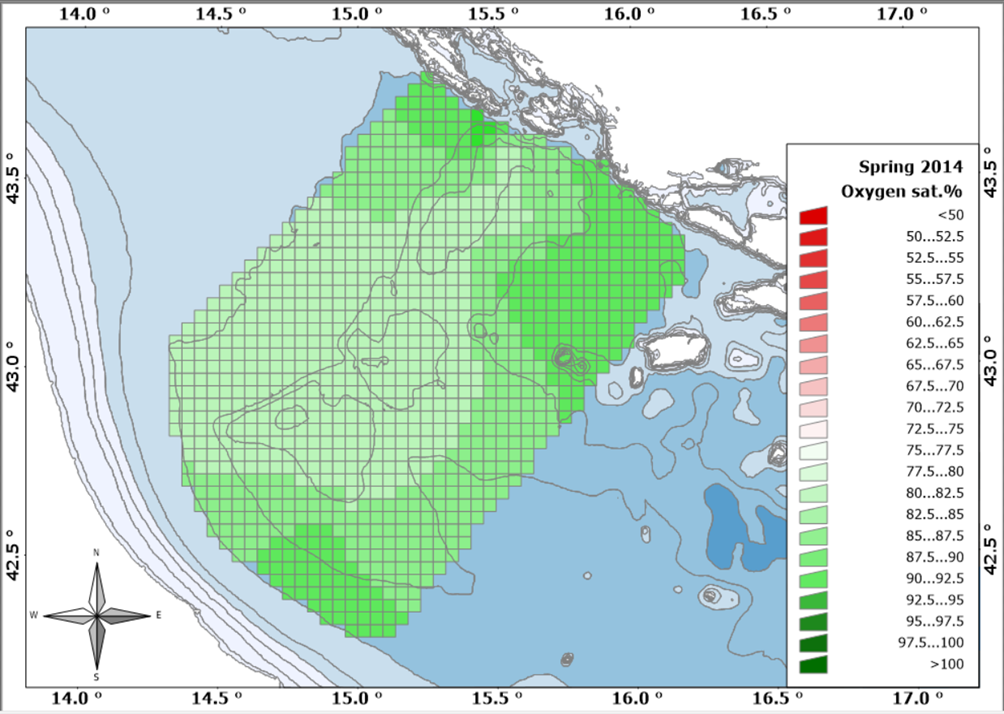


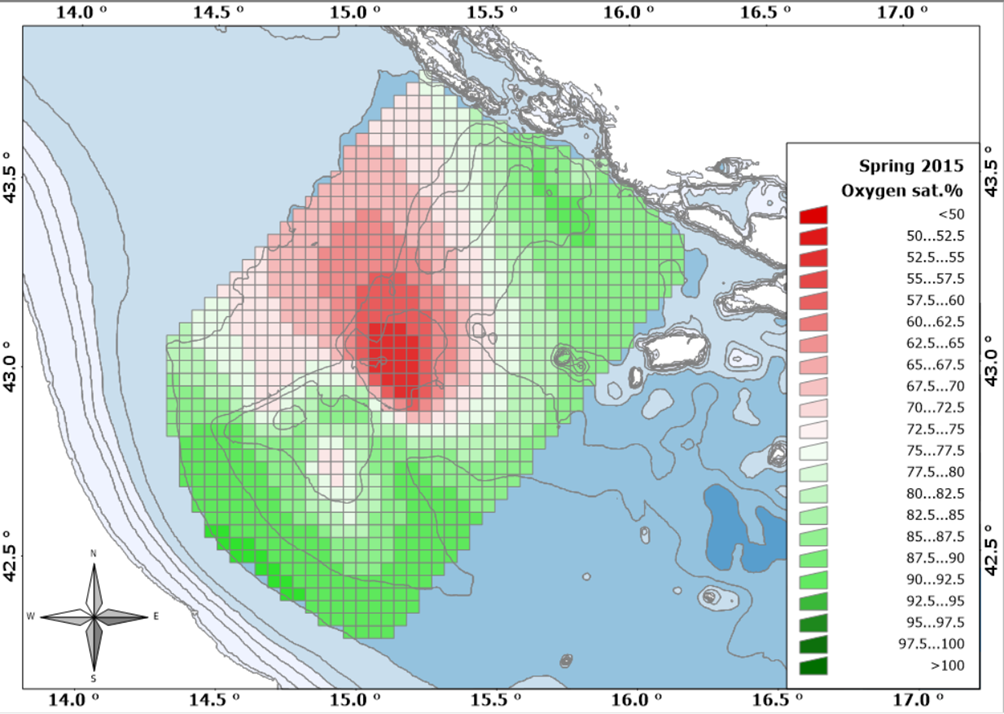


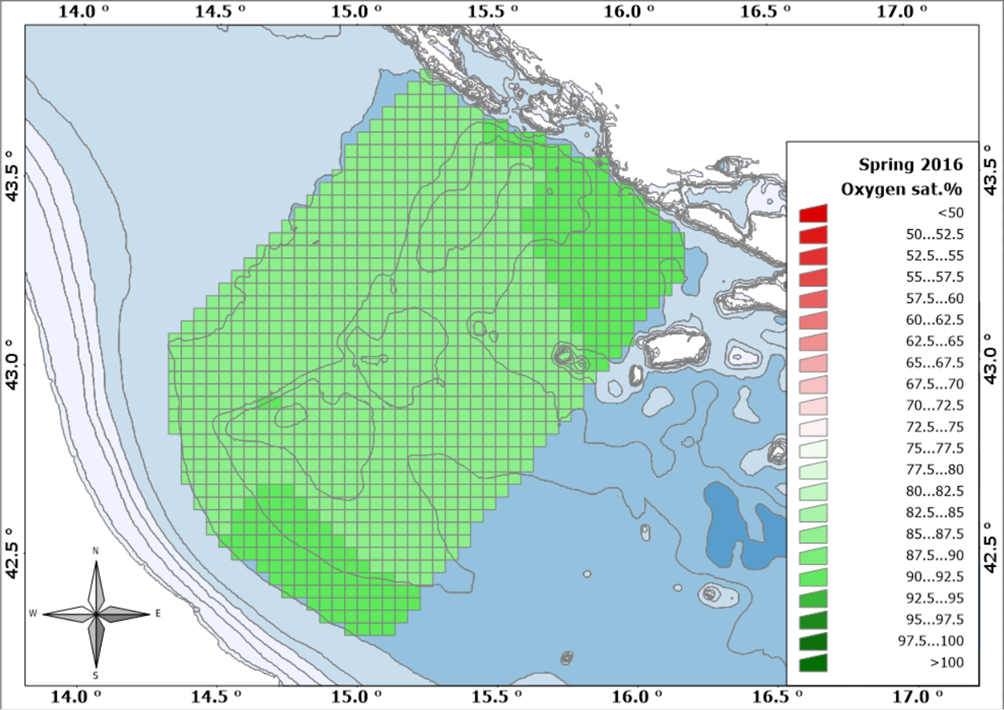


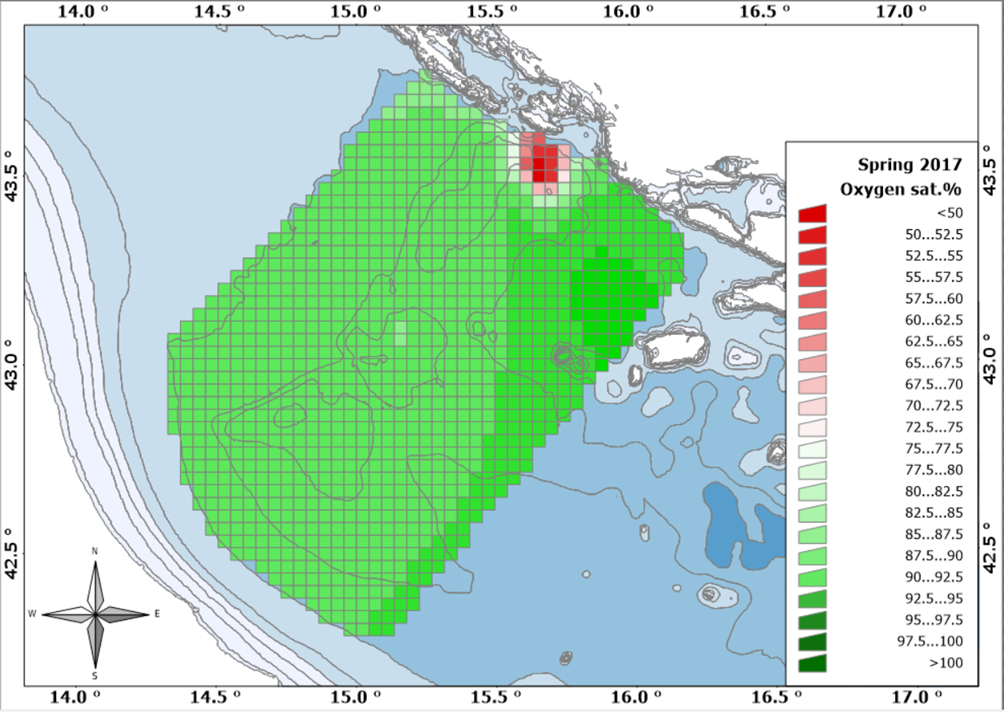


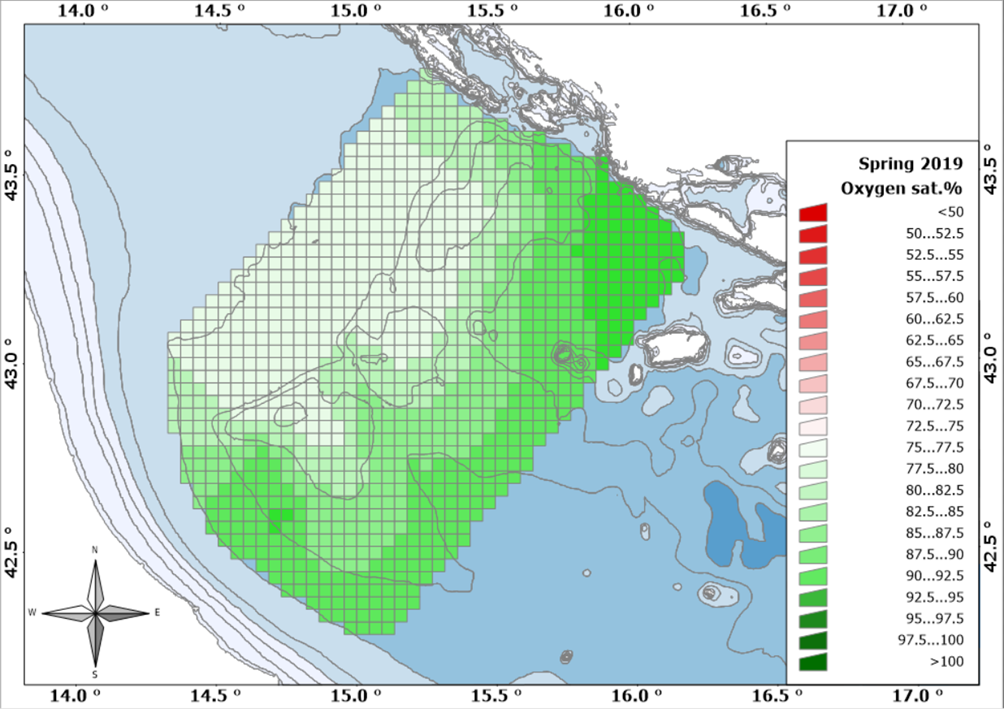


**S9 Fig. Maps of bottom dissolved oxygen saturation percentage for the autumn time series.** Bathymetry layer source: [75].


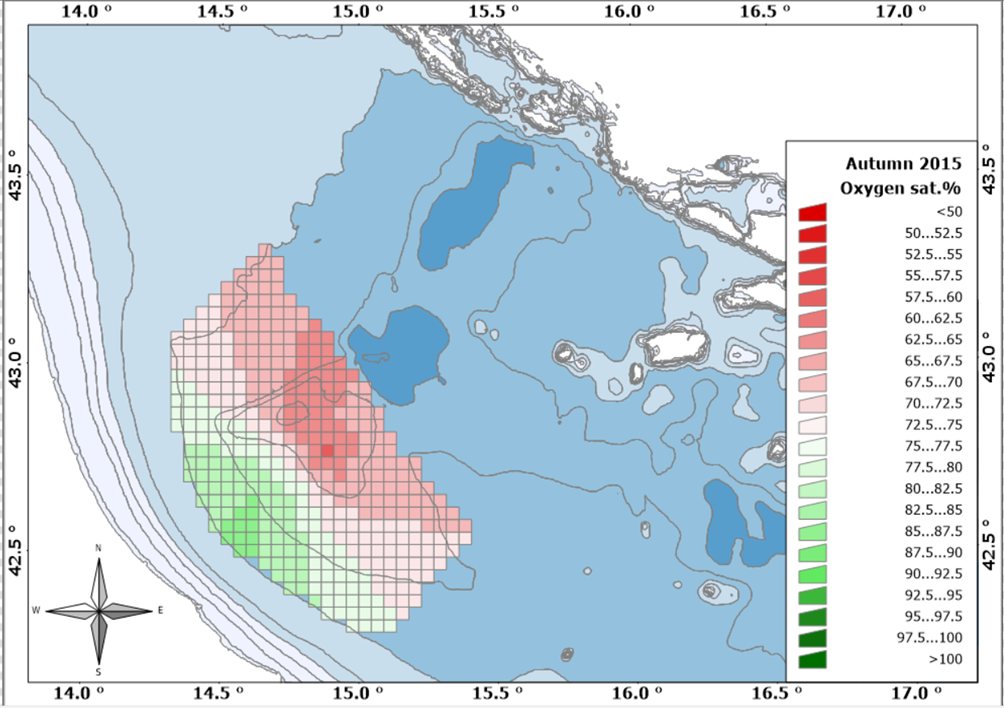


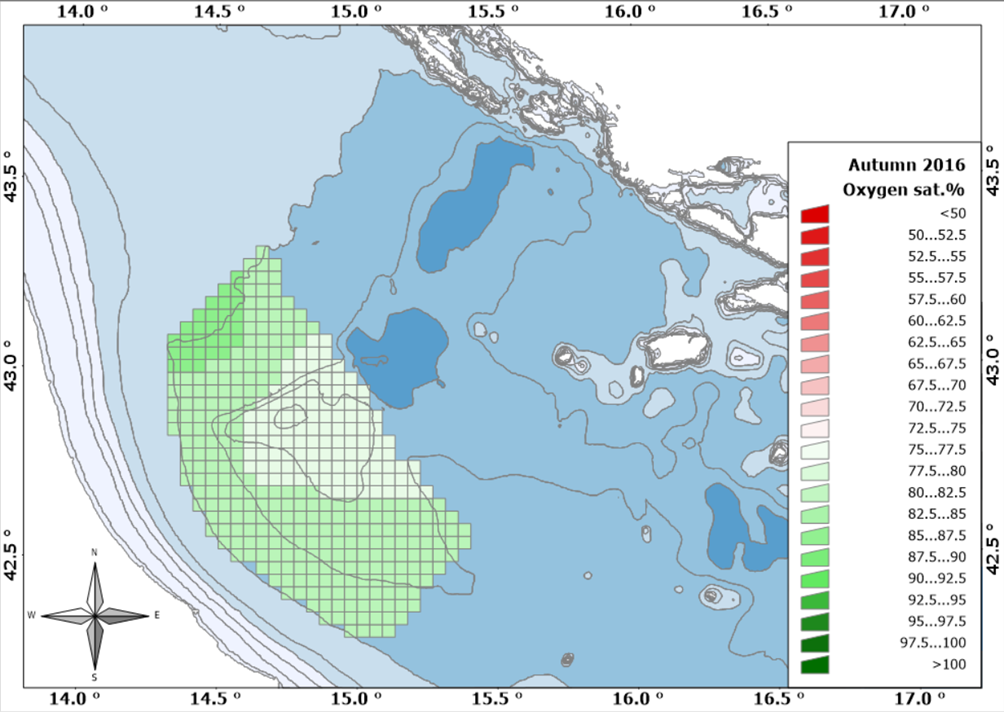


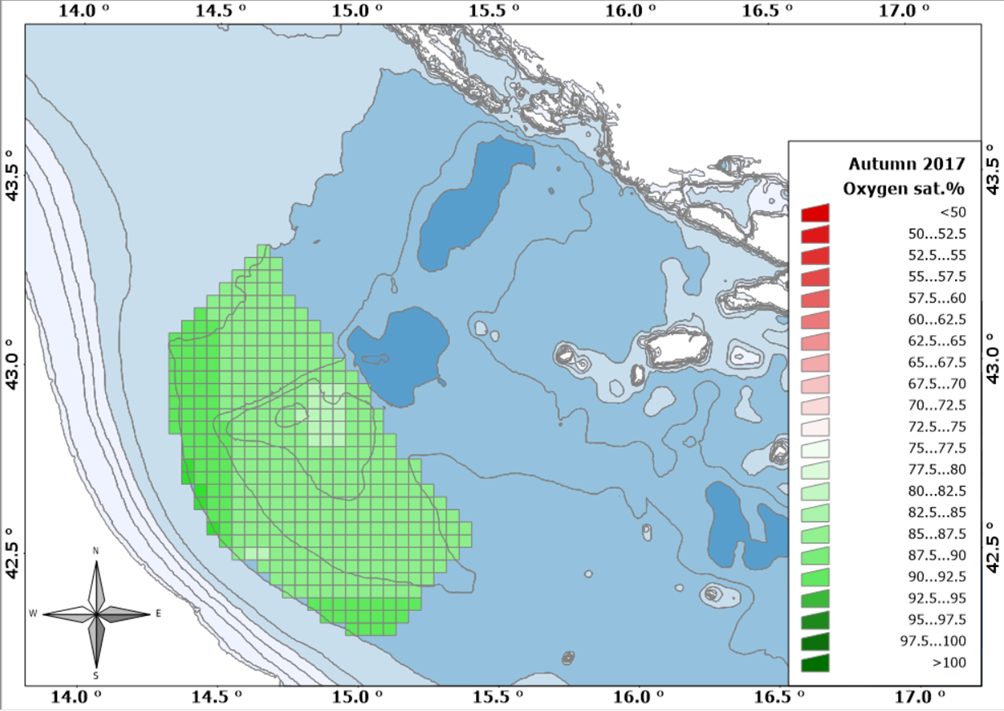


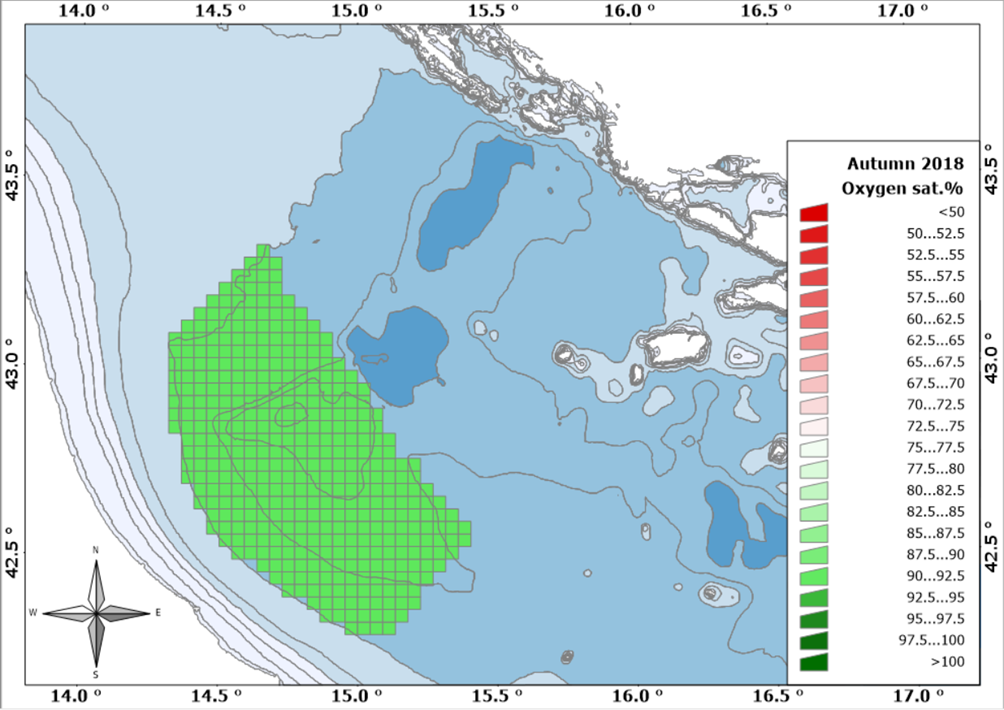


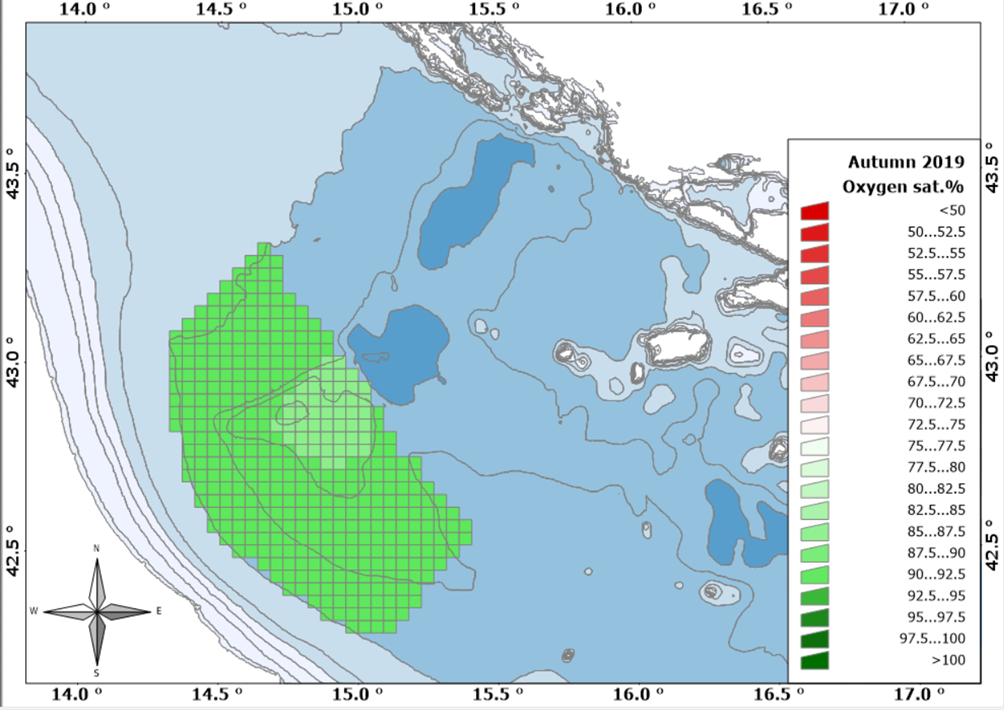


**S10 Fig.** **Maps of bottom temperature values for the spring time series.** Bathymetry layer source: [75].


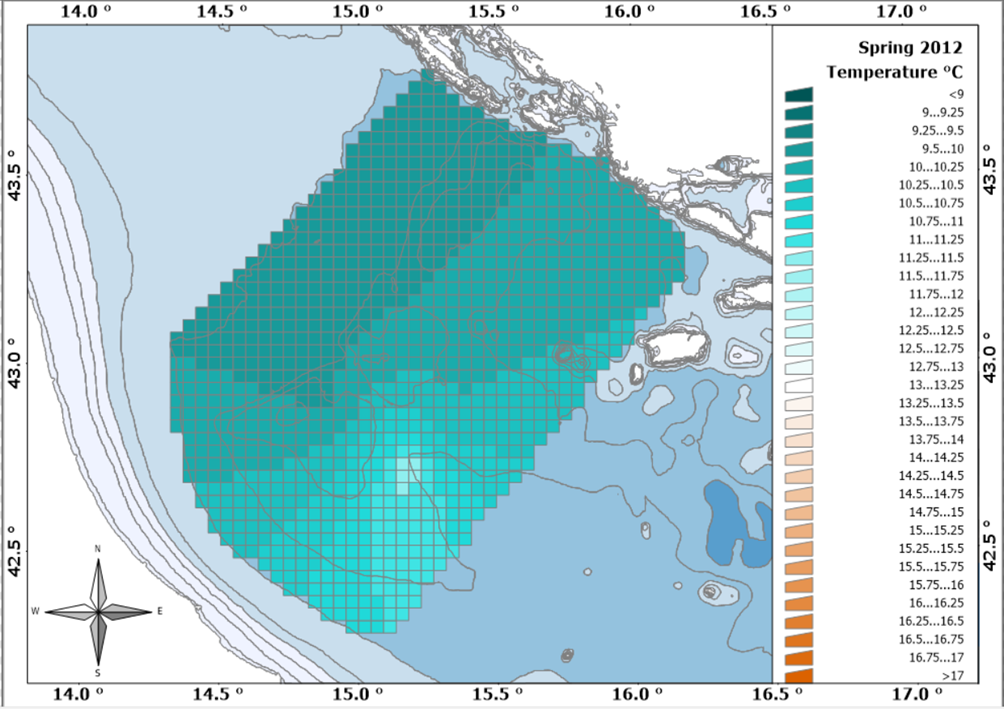


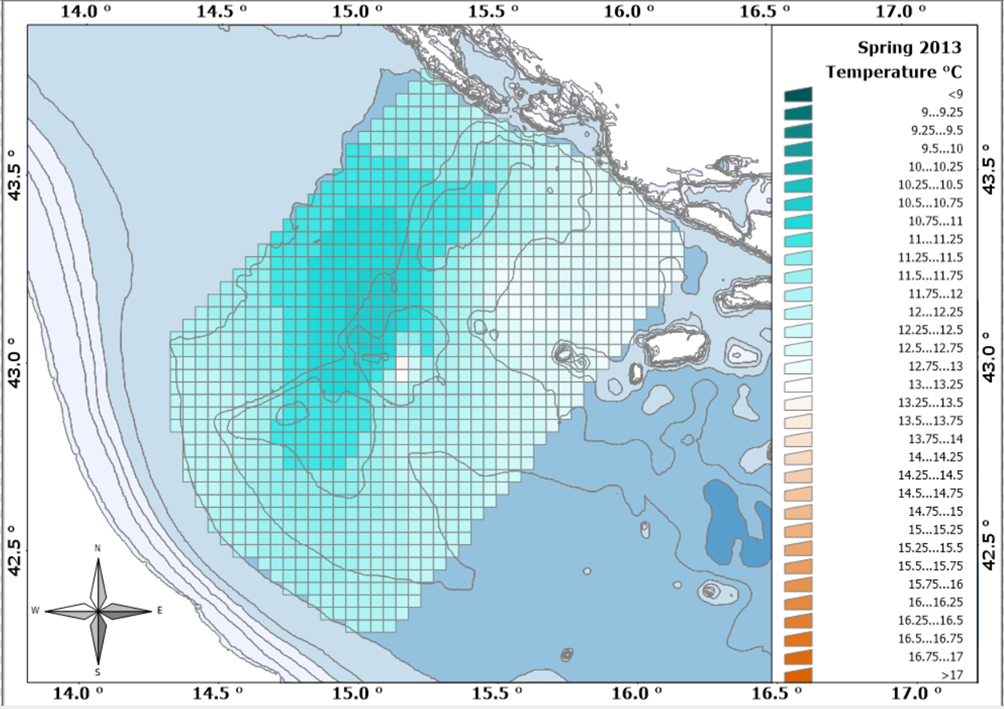


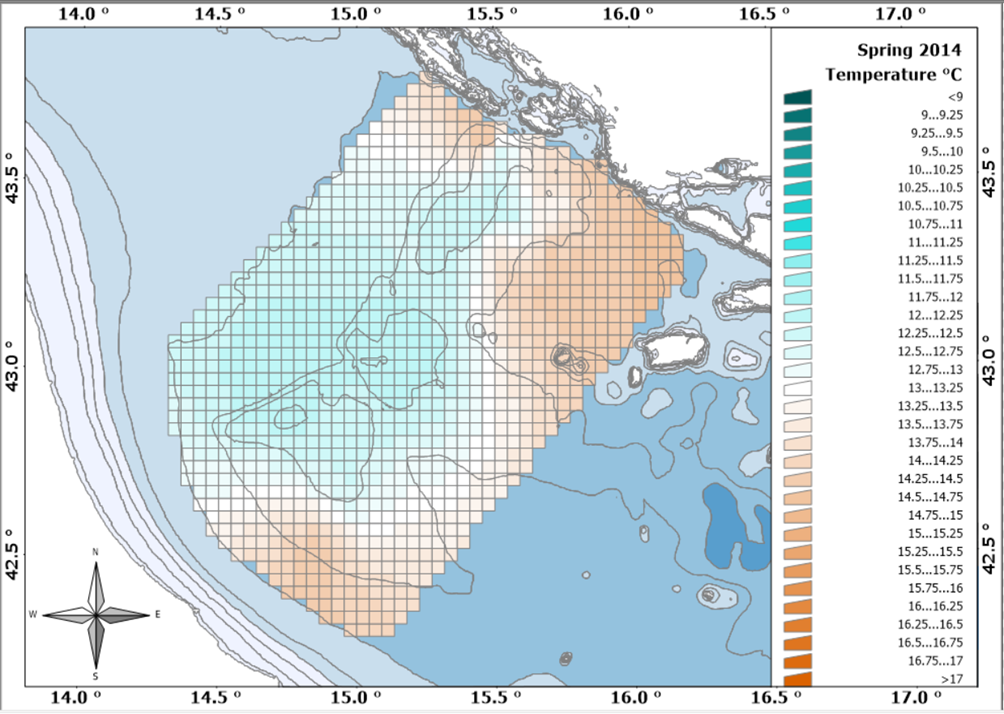


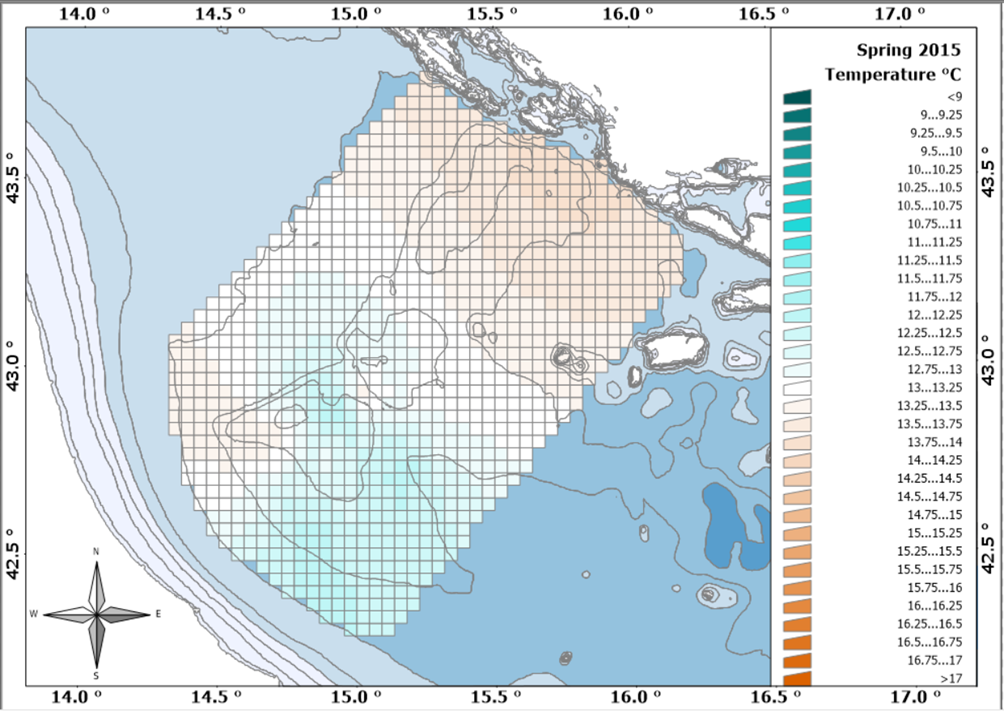


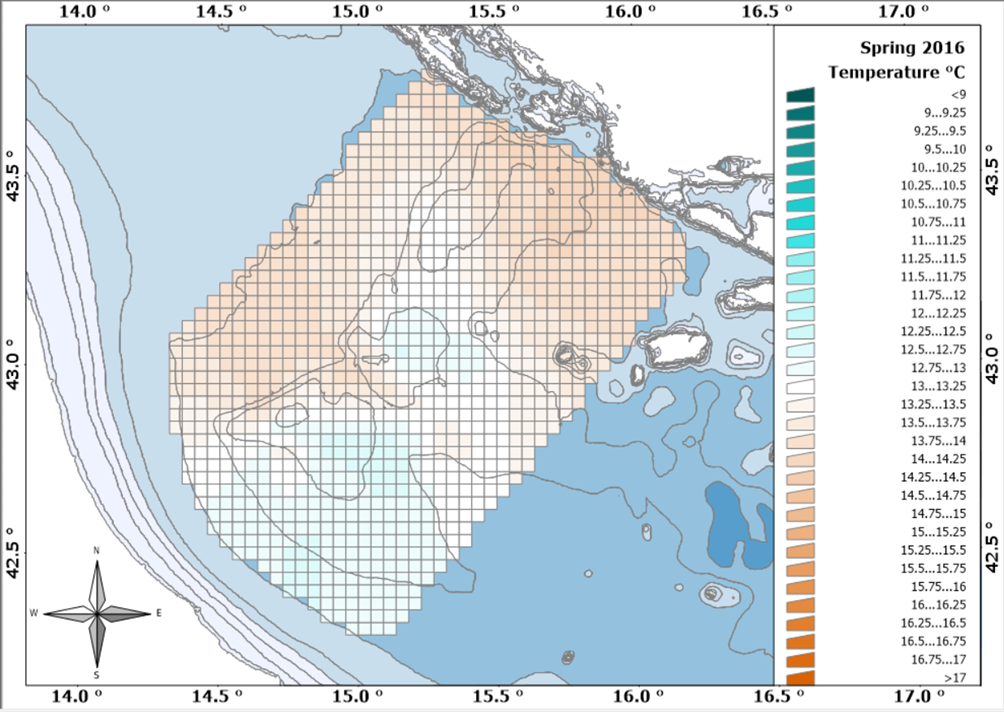


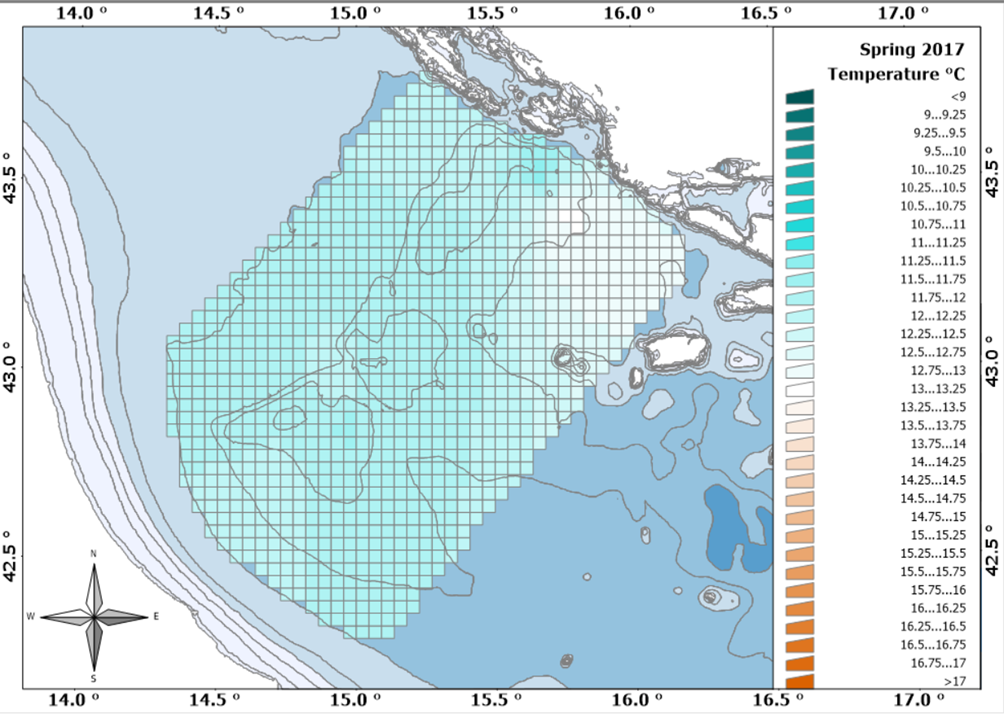


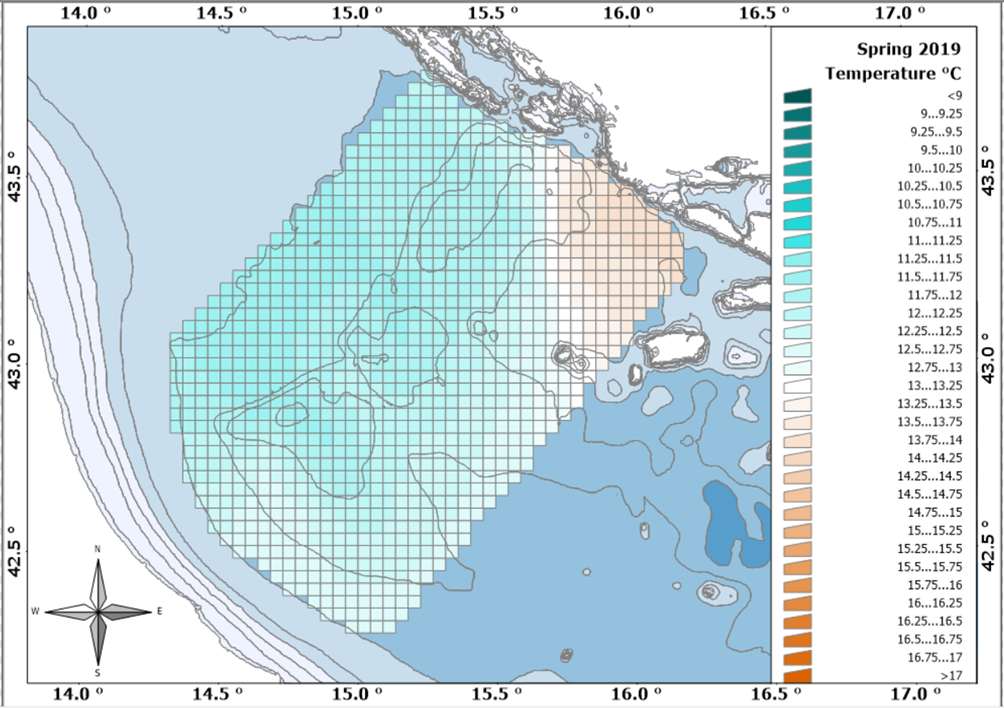


**S11 Fig. Maps of bottom temperature values for the autumn time series.** Bathymetry layer source: [75].


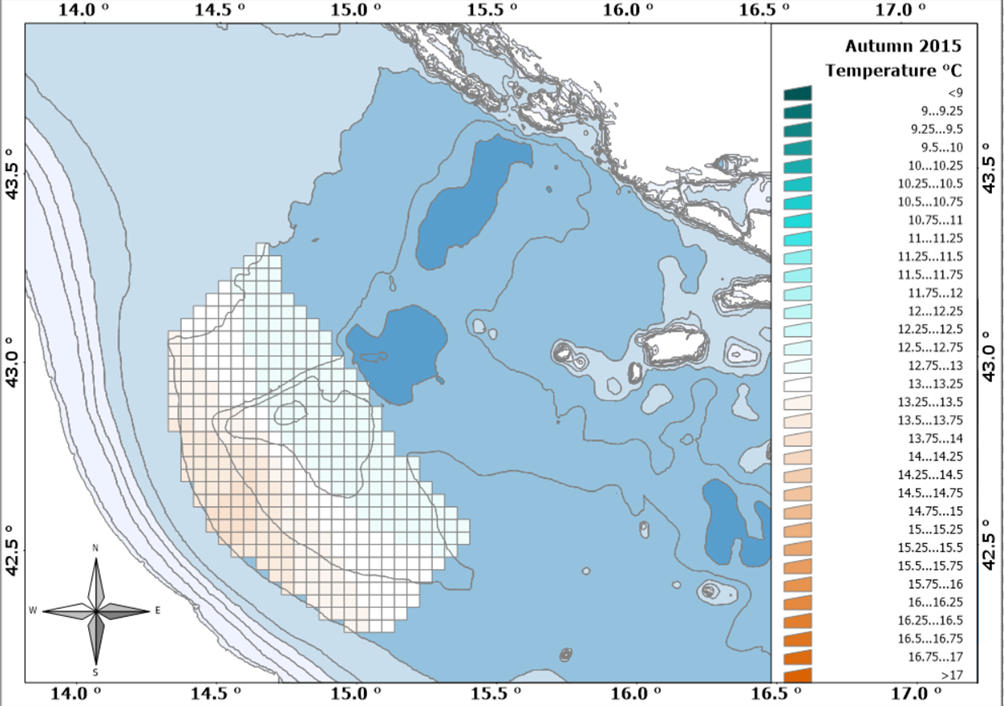


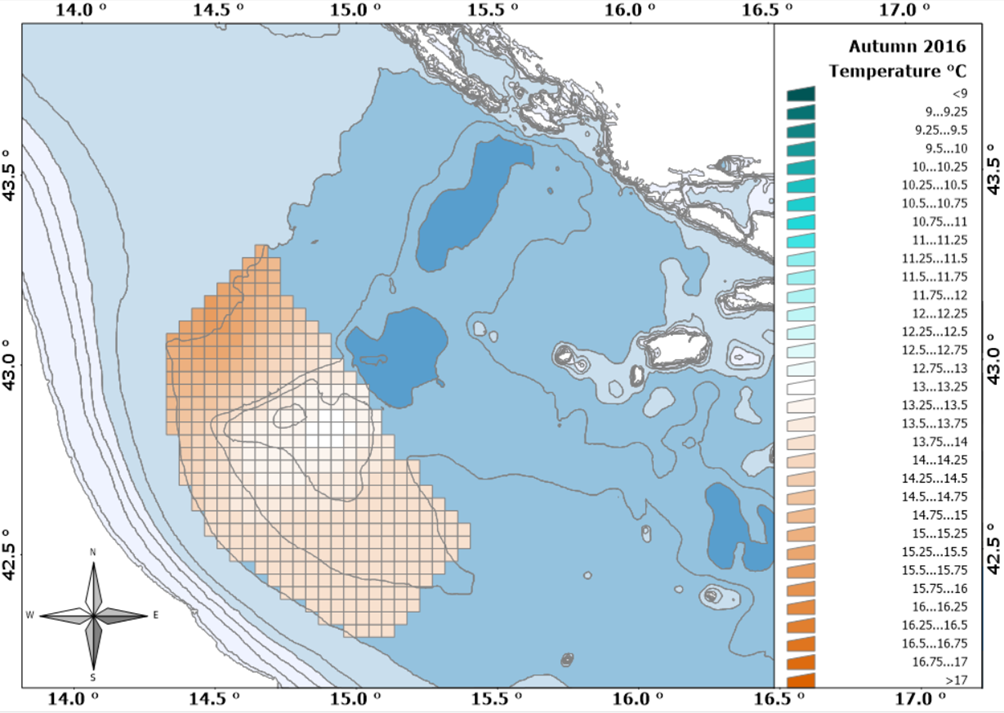


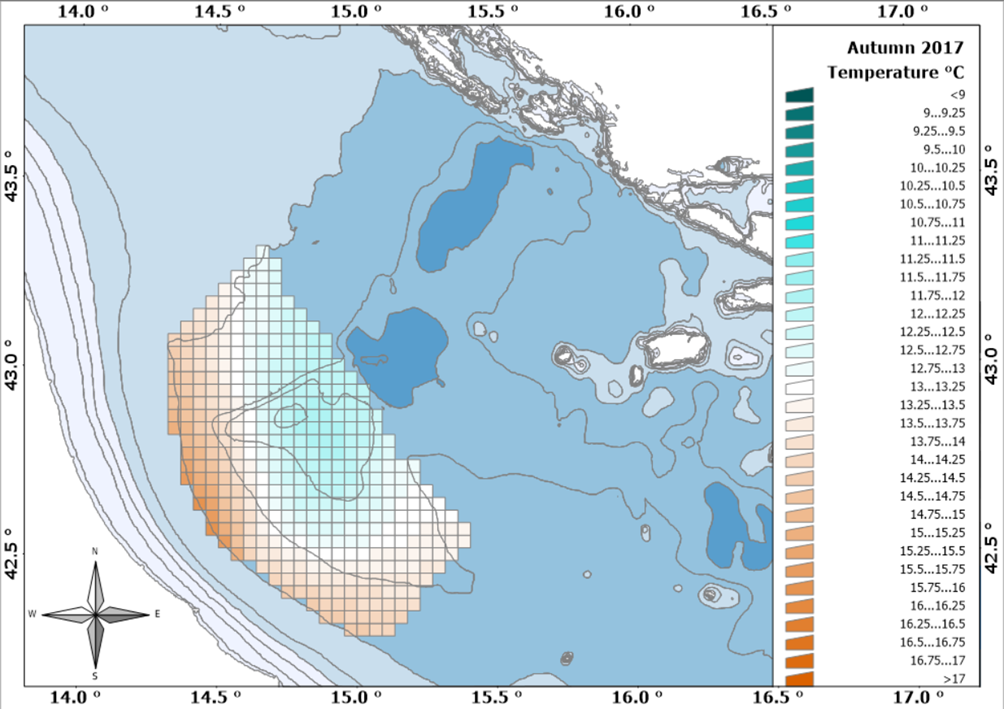


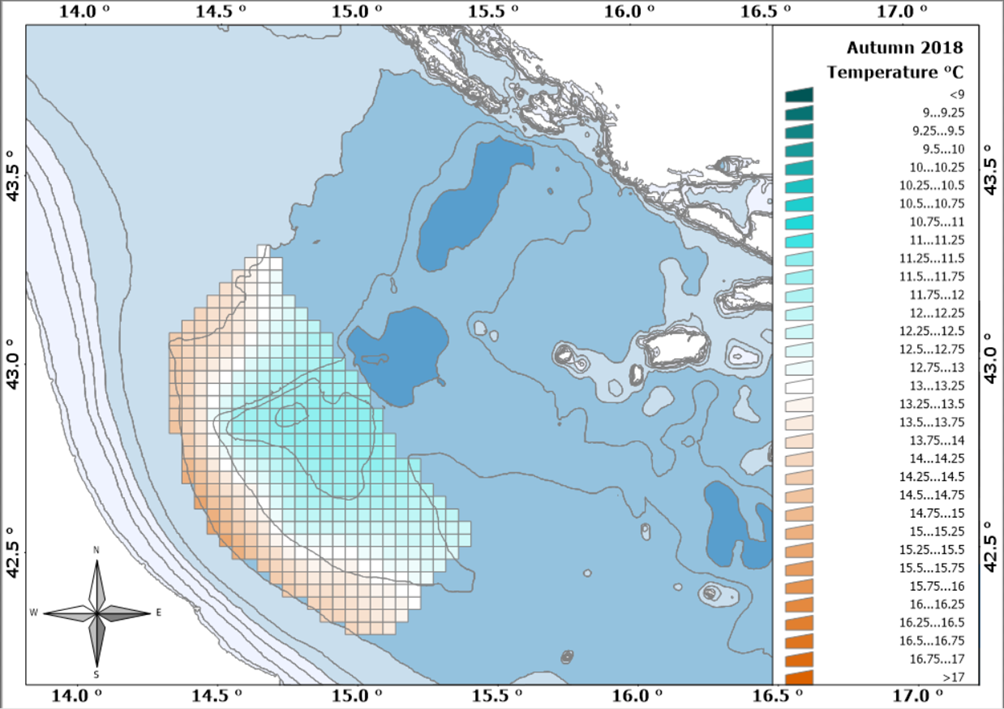


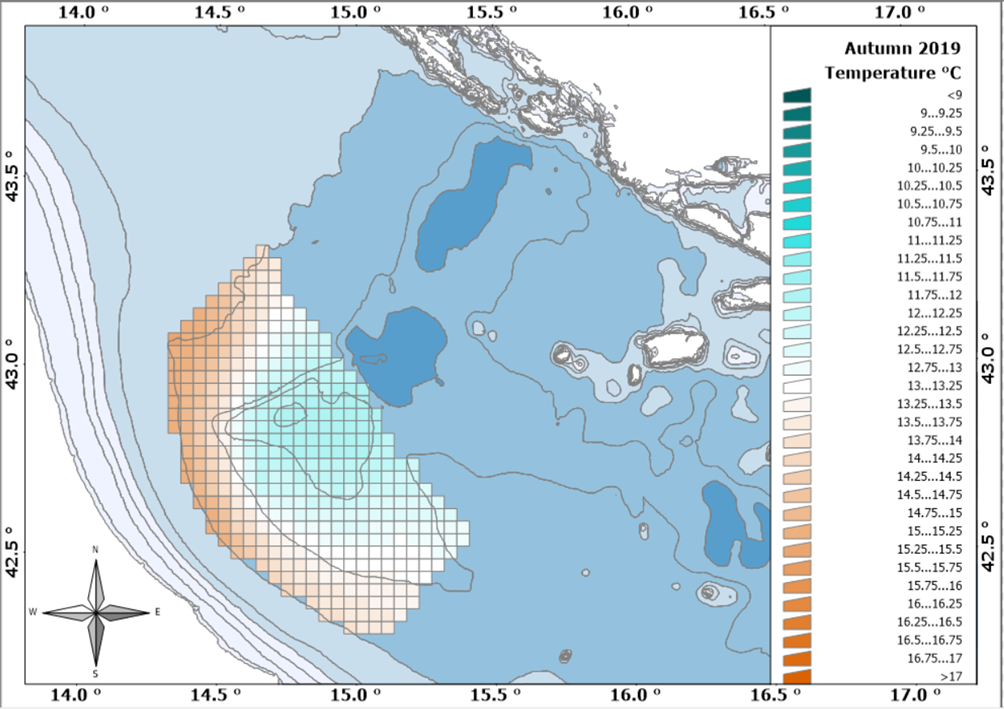


_______________________________________________________________


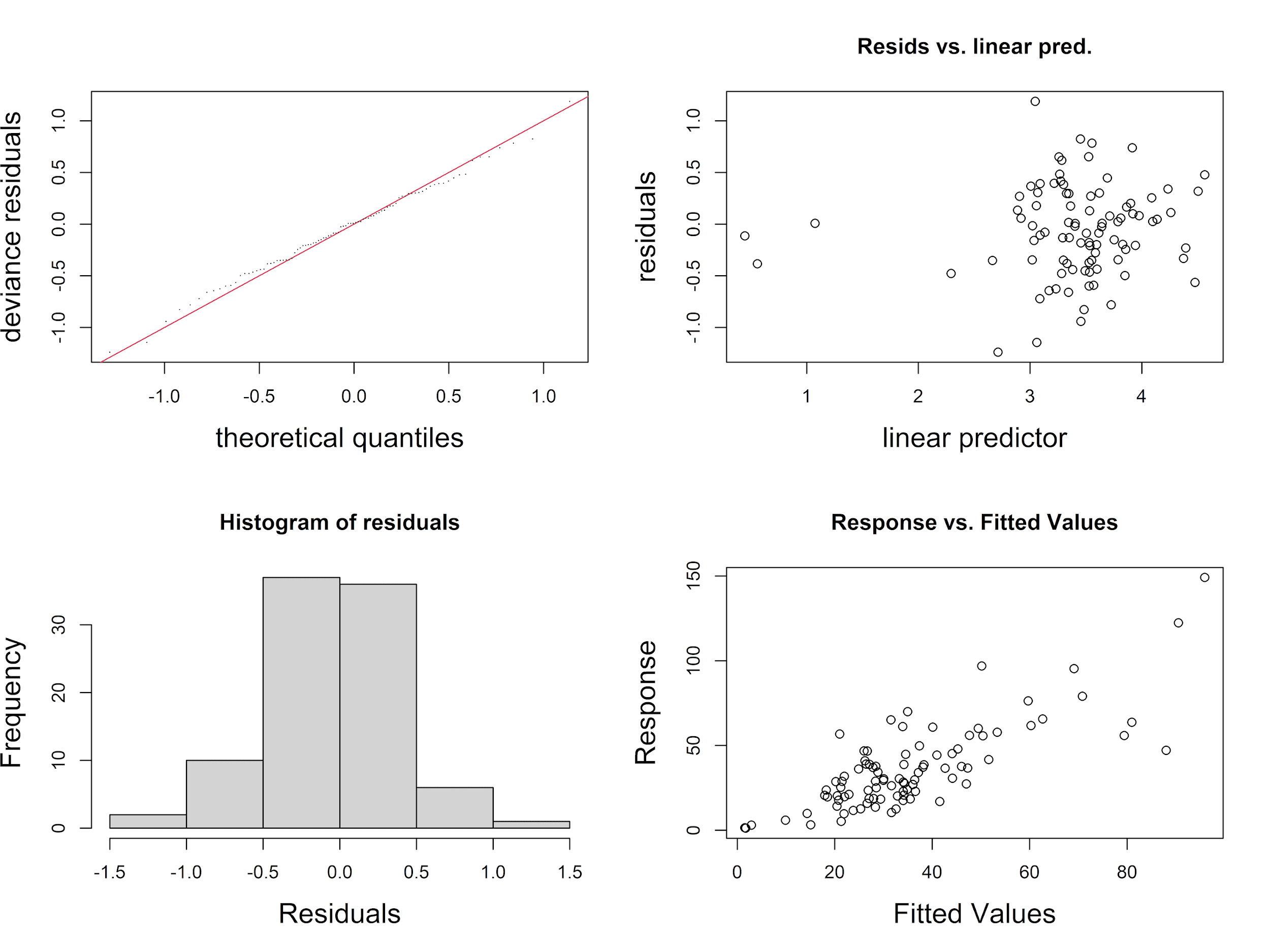


**S12 Fig.** **Residual analysis for the final GAM for biomass index.** Q-q plot, histogram and dispersion of residuals show mean around zero and homogeneity of variance.


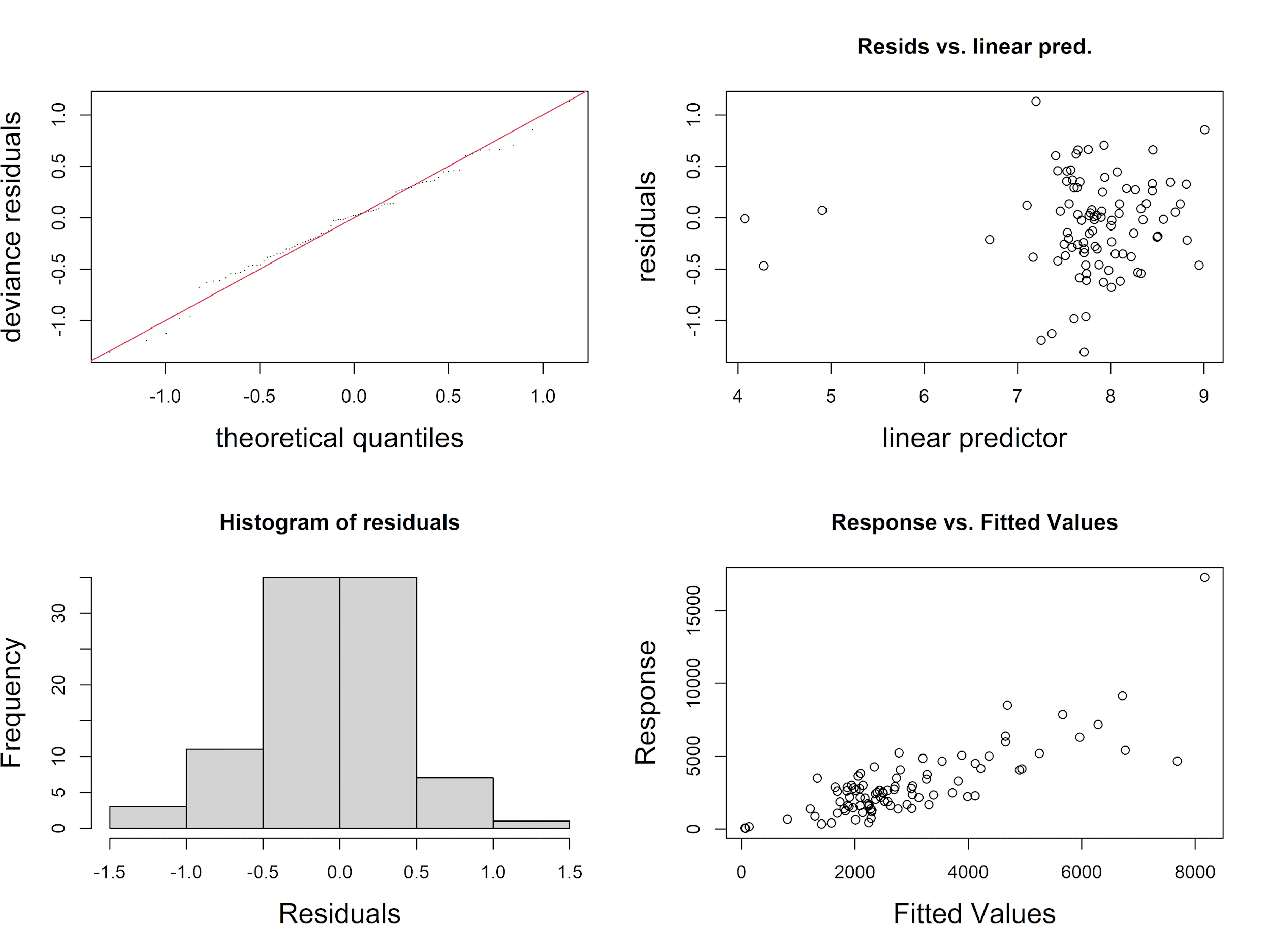


**S13 Fig.** **Residual analysis for the final GAM for density index.** Q-q plot, histogram and dispersion of residuals show mean around zero and homogeneity of variance.


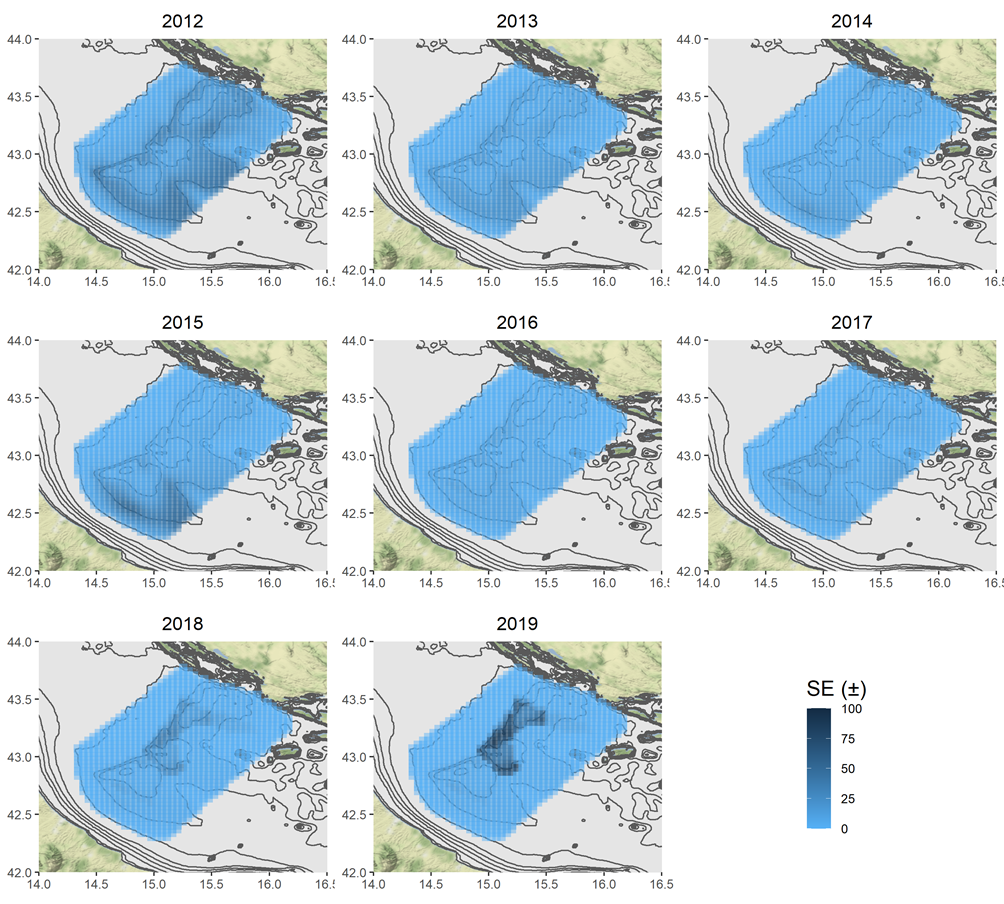


**S14 Fig.** **Predicted standard errors of *Nephrops* biomass index (kg/km^2^) for the spring time series.** Maps were made using the ggmap package [105] for R. Bathymetry layer source: [75]. Map tiles by Stamen Design, under CC BY Data by OpenStreetMap, under ODbL.


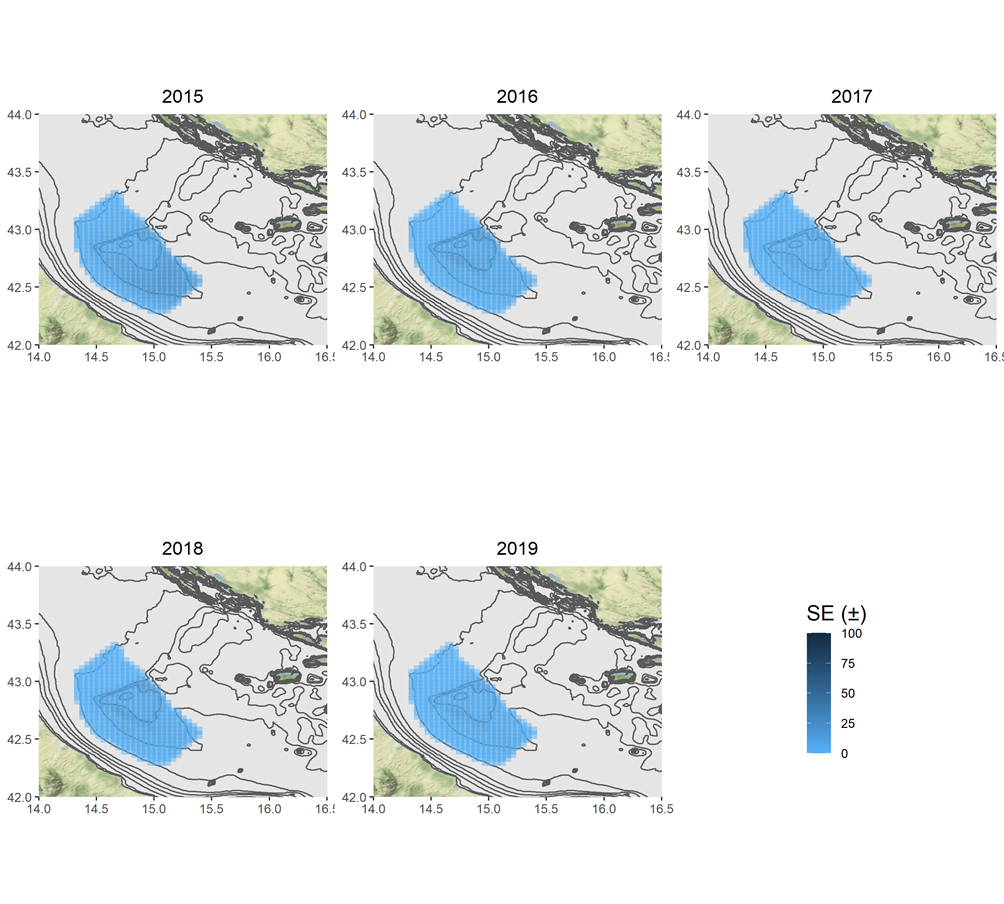


**S15 Fig.** **Predicted standard errors of *Nephrops* biomass index (kg/km^2^) for the autumn time series.** Maps were made using the ggmap package [105] for R. Bathymetry layer source: [75]. Map tiles by Stamen Design, under CC BY Data by OpenStreetMap, under ODbL.


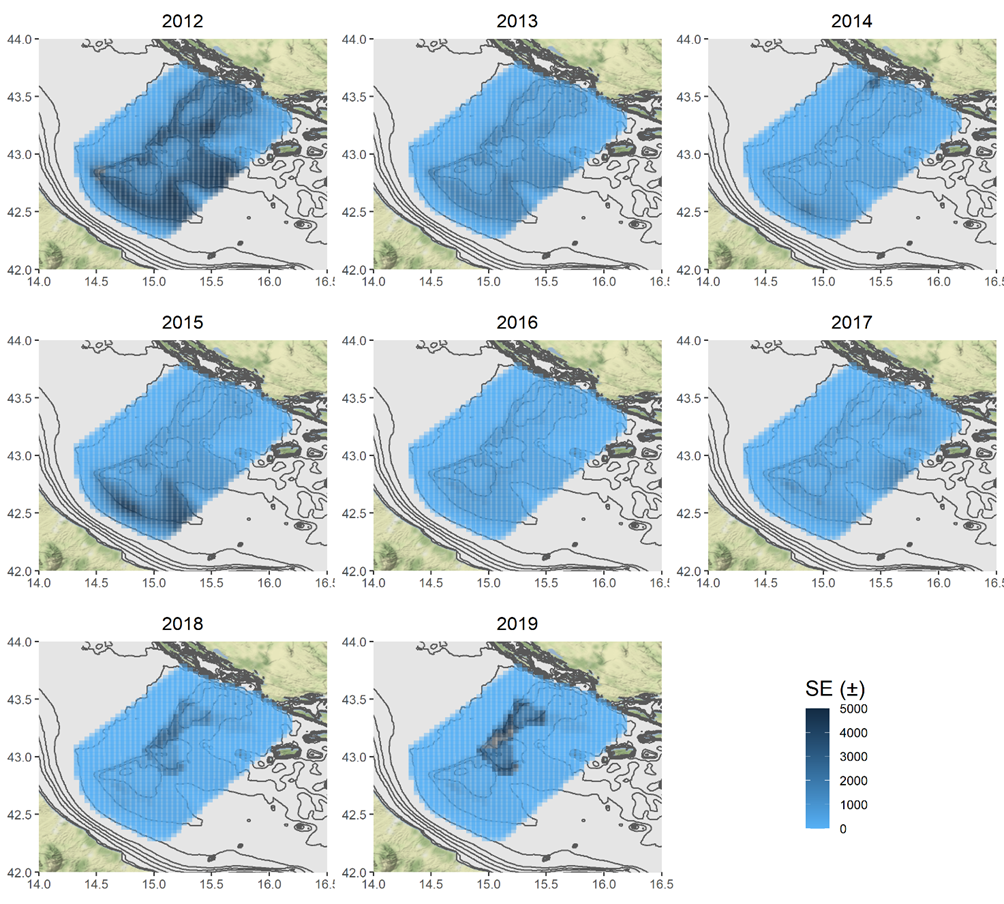


**S16 Fig.** **Predicted standard errors of *Nephrops* density index (N/km^2^) for the spring time series.** Maps were made using the ggmap package [105] for R. Bathymetry layer source: [75]. Map tiles by Stamen Design, under CC BY Data by OpenStreetMap, under ODbL.


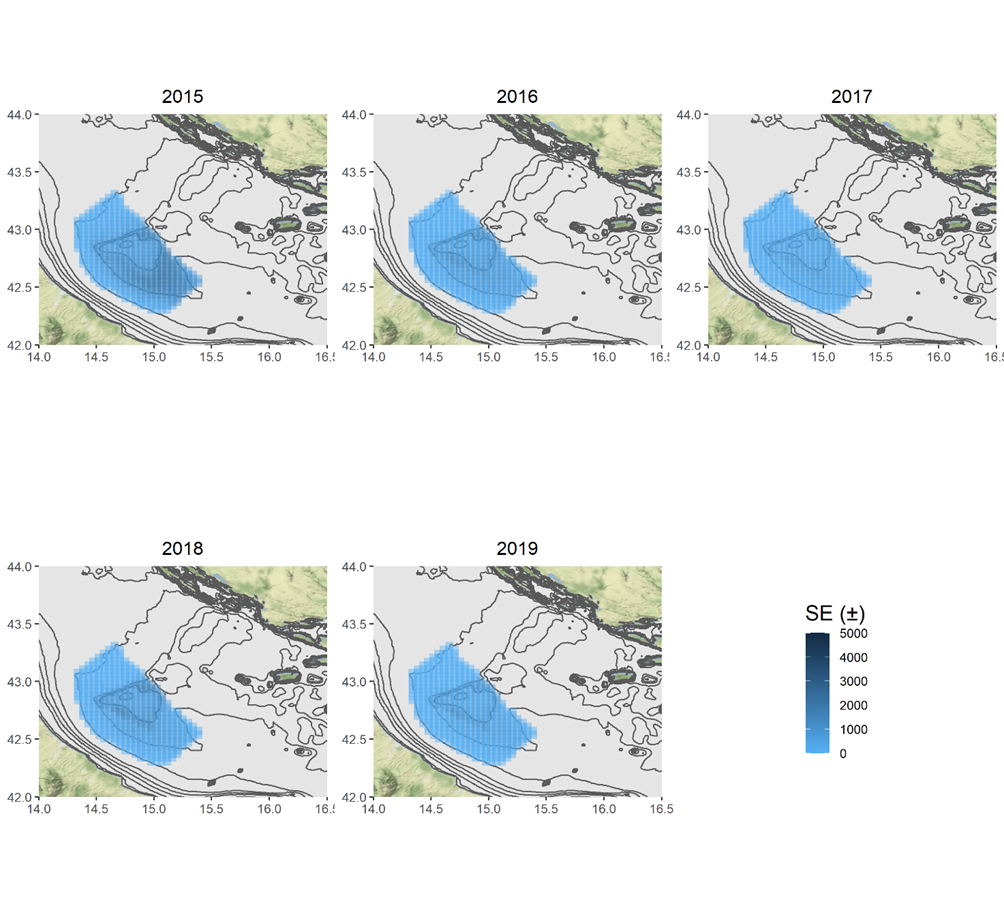


**S17 Fig.** **Predicted standard errors of *Nephrops* density index (N/km^2^) for the autumn time series.** Maps were made using the ggmap package [105] for R. Bathymetry layer source: [75]. Map tiles by Stamen Design, under CC BY Data by OpenStreetMap, under ODbL.
