# Supplementary material for: Accounting for environmental and fishery management factors when standardizing CPUE data from a scientific survey: A case study for Nephrops norvegicus in the Pomo Pits area (Central Adriatic Sea)
Source: PLoS One. 2022 Jul 14;17(7):e0270703. doi: 10.1371/journal.pone.0270703 (PMC9282463; doi:10.1371/journal.pone.0270703)
Supplement: Attachment — Submitted filename: reviewers comments_FINAL.docx [file pone.0270703.s002.docx]

Reviewers' comments to submission PONE-D-21-25011

Author's Responses to Reviewers' comments were written in red characters

**Comments to the Author**

Reviewer #1: # Overall

The study presents an analysis of Norwegian lobster CPUE values from scientific surveys, using GAM models including spatial, temporal and environmental explanatory variables to standardize CPUE values. Though the writing is generally clear and detailed and the work appears sound on a technical level, I have some concerns and doubts about the underlying assumptions of how the standardization was carried out that are detailed below. I recommend major revision and publication only if these issues can be addressed or clarified.

\# Major comments:

My most significant question regards the treatment of environmental and spatial variables when estimating standardized CPUEs. It is not clear from the methods whether environmental variables were held constant when predicting standardized CPUEs (and therefore considered to impact only catchability, and not abundance) or if they were allowed to vary freely over space and time (and therefore considered to impact only abundance and not catchability), though it would seem from Fig. 5 that the latter choice has been made.

The environmental variables were free to vary over space and time within a grid of the Pomo/Jabuka Pits area replicated for each survey/year combination. Therefore, the environmental variables collected during the surveys were interpolated by kriging method resulting in a mean value for each cell of the grids. The environmental maps adopted in this study have been added in the supplementary material for each year/survey (Supplementary Figures S5-S10), and the grid building process was better specified in the material & methods section

Without knowing the details of this species or fishery, it is hard to say which if either of these extremes is most appropriate, but in general environmental variables are a little bit of both making their treatment in CPUE standardization particularly complicated. Would it be better to separate each environmental variable into the longterm running average over the typical lobster lifespan and the anomaly with respect to this running average, interpreting the first as an indicator of abundance (i.e., habitat suitability) and the latter as an indicator of short-term changes in catchability (e.g., due to changes in animal behavior in response to immediate environmental conditions)? If not, please clarify what motivates treating these environmental variables as only impacting catchability or only impacting abundance. Also, if the choice has been made to let environmental variables vary over space and time, isn't there a problem of temporal scale in that longterm trends in environment iver the lifespan of an individual likely determine abundance, whereas your model appears to use immediate environmental values.

Within this study the retained environmental variables were collected during the surveys, before or after each haul, as close as possible to the fishing location. The collected point-like values were interpolated through kriging method to build grids for each survey and year resulting in average environmental values for each cell of these grids (the related maps have been included in the supplementary material and more details in the text have been provided). The reason behind the use of environmental parameters collected directly from the surveys is due to the difficulty of finding reliable bottom environmental data especially at the season/year level. The environmental parameters used in this study were the best that could possibly be obtained in order to investigate their effect on *Nephrops’* distribution (intra-seasonal spatial variability) and its catchability (seasonal variability caused by *Nephrops*’ emergence behaviour). The significance of each of the environmental variables retained in the GAMs was calculated and their effect on the spatial and temporal CPUEs distribution was discussed in the results and discussion sections. Moreover, within the discussion, the possibility of a future introduction/replacement of environmental terms with those related to earlier periods was stated; the aim would be to investigate the effect of environmental variability on different life stages, for example modelling the recruitment with salinity values recorded years before. We appreciate your suggestion to also try to use averages on lifespan. It’s certainly interesting but it’s an approach that wasn’t considered in this study, however a reference to this approach has been added.

Similarly for spatial variables it is not 100% clear if predictions for each grid cell were simply averaged when estimating annual time series in Figure 4, though presumably this is the case. In general, please clarify which variables were held fixed and which variables varied over space and time and how averaging was performed over space and/or factorial variable levels when making predictions.

The annual time series estimated by means of the final model (eq. 4) shown in Fig 4 corresponds to the average of the predicted values for each cell within the considered seasonal domain. Only the covariate “ToD” was fixed at “sunrise”; the depth for each cell was held constant because the bathymetry layer used to build the grid was the same for each year/survey combination. while all the other variables vary for each cell over space and time.

GAM formulation: week appears to be modeled as a standard smooth (bs="tp"), but wouldn't it be more appropriate to use circular basis functions (bs="cc")?

All the terms in the model were modelled as penalized cubic regression splines (bs=”cr”); in accordance with both reviewers the spline for the term “week” was changed with a circular basis function (bs=”cc”) and all the tested models were run again improving all the performance metrics.

Figure 2: There seem to be quite a few anomalous values along the X axes of some of the plots that the model is having a hard time fitting. What do these correspond to? Zero densities? I do not see strong evidence of these anomalies in the model diagnostics in Figs. S1 & S2, but the QQ plots are very hard to see (this should be fixed) and the residuals vs. linear predictors plots does seem to show heteroscedasticity with perhaps all of these anomalous values superposed on the left side of the graph? This should be clarified and corrected if need be.

The values on the X axis in figures 2 and 3 are not zero density but they reflect the distribution of the data along the variable (i.e. the relative density of data points, commonly called “Rug plot”); this is useful to understand if the data are aggregated for a certain value of the variable or they are homogeneously distributed throughout the range of possible values of the selected variable. This should have been explained in the captions; however, this information has been removed from the plot and its resolution has also been improved in order to make it more understandable and readable.

\# Minor comments:

Line 32: Shouldn't this be "fishery-dependent data"? I see now why you
say "fishery-independent", but it would be less confusing to say
scientific survey data as CPUE standardization is most often applied to
fishery dependent data.

Changed in “scientific survey”

Line 39: "peculiar" is hard to interpret. "particular"? "irregular"?
"unstable"?

“particular”

In the abstract, the authors pose an important question - how to
address the impact of environmental variables on catchability and
abundance in a species. But the Abstract ends without ever really
providing an answer to this question. How did the predictive grid help
you answer this question? How can the predictions be used to better
describe distributions?

Some sentences were entered in the abstract accounting for both questions. In particular, this new version of the abstract states that GAMs can be used to understand the impact of significant drivers on CPUE in order to remove most of the variability. Focusing on environmental and fishery covariates, the effect of the independent variables on spatial distribution and abundance was thus investigated by GAMs; if they significantly influenced the dependent variable, they were included in the standardization process by means of predictive grids. Grids for each survey and year were thus built aiming to predict the spatial distribution of CPUE according to the values contained in each cell. Once the final model was built and tuned on all the data at hand, it was able to predict the spatio-temporal distributions of standardized CPUE for each cell of the grid accounting for both the effects of environmental parameters and fishery management measures in place.

Line 62 and before: CPUE is impacted by fishery technological or
strategy changes. Also, CPUE is not just used for crustaceans, but
also for essentially all exploited marine species for which scientific
survey data is impractical to obtain or unreliable.

Sentence adjusted

Line 69: "other than abundance [8] and survey operational shortcomings" is confusing as it makes it sound like we want to remove everything BUT abundant AND survey operational shortcomines.

“survey operational shortcomines” was removed and the sentence was changed in “it would be relevant to remove factors other than abundance that may influence CPUE variability before using an index as an indicator of population size”

Line 74: " missing observations is systematic" - I would have said "random" or "unpredictable", more or less the opposite. If certain areas are systematically not sampled, then no amount of standardization can fix that.

The sentence was changed to better specify that an adequate pattern of observations at the appropriate spatial scale may lack during scientific surveys.

Line 86: "such as"

Added

Line 121: "despite" doesn't make sense here. "highlighting"?

Changed in highlighting

Line 157: You state that the habitat is ideal, but the individuals are "smaller", which seems counterintuitive to me. Please clarify.
As stated in the introduction, *Nephrops* is featured by spatially segregated populations with different densities and length structures; however it has preference for muddy grounds allowing the formation of its characteristic burrows, for that reason the study area was defined as ideal habitat. A sentence has been added to recall these aspects.

Lines 174-185: These sentences are run-on and incredibly dense. Break into several sentences. In general, this paragraph has too many parantheses, making it hard to read.

The paragraph was reformed.

Line 209: "was" -> "were"

Changed

Line 216: This seems like quite a bit of data that was excluded. Perhaps you could include a table identifying the years and localities of excluded data? Is there any indication of spatial or temporal bias in exclusions that might be important for results?

The mean of removed stations by year has been calculated and more details about the temporal distribution of excluded hauls have been provided.

Line 221-222: Are you saying the data are not normally distributed because they are positively skewed? Clarify.

Exactly! This aspect is now better specified in order to clarify the sentence.

Line 223: "they" would seem to refer to several outliers, but then a single density is indicated, leading to confusion. Please clarify.

Here only the highest values are specified. “They” was removed.

Line 225: You might want to say that a Gamma distribution is a positive-definite two-parameter distribution that converges on a normal distribution for small CV.

The sentence was adjusted in order to better explain that a Gamma distribution with logarithmic link function works well for positive-only data. Moreover, the Gamma distribution is one of the most used GAM families for modelling environmental analysis as stated in Ofungwu (2011).

Ofungwu J., Statistical applications for environmental analysis and risk assessment, Wiley, 2014

Line 248: You talk of the "full model", but then it is also stated that interaction terms were added to the model. It would probably be more useful for the "full model" to be the superset of all models examined, thereby including both direct and interaction terms.

Actually, the one previously defined as the “full model” has been revised in this second version of the submitted paper; the “full model” equation called “mod INITIAL” includes all available terms both as single and interaction effects which is supposed to best explain the variability of the response variables being the most complete (i.e. containing the maximum number of available information), as described in Zurr et al. (2009). The “full model” (or initial) has been then tested against models excluding, on the one hand, the effect of fishery management actions and, on the other hand, the effect of environmental variables in order to assess whether these information effectively increase the performance of the model.

Line 304: What was X correlated with and why did you choose to exclude X instead of the one or more other variables it was correlated with?

X was correlated with all the other variables (it’s a multicollinearity analysis) and was excluded instead of other variables on the basis of the resulting VIF value (VIF > 3).

Line 307: "with the best accuracy was the same"

Changed

Line 351: "of" -> "by"

Changed

Lines 391-392, lines 428-420, lines 447-448: These sentences do nothing more than point out the relevant figures and can be deleted. In general, text should present results rather than just presenting figures. Lead with what you want the reader to know about the contents of these figures.

The sentences were adjusted pointing out the contents of the corresponding figures.

Line 483: "scales"

Changed

Line 567: "consequent"??

Changed in “caused by”

Reviewer #2: The manuscript "Accounting for environmental and fishery management factors when standardizing CPUE data from a scientific survey: a case study for Nephrops norvegicus in the Pomo Pits area (Central Adriatic Sea)" faces an interesting field. Although the dataset collected is very interesting because it comes from two seasonal surveys in an area subjected to different management measures, the manuscript presents several methodological gap and inconsistencies.
The authors present a standardization of such CPUE time series by mean of Generalized Additive Models aiming at investigate how the environmental variables and the different management strategies can influence abundance and distribution of the target species in the study area.

The introduction section is confused and do not guide the reader to understand completely what the standardization process consists of.

In the introduction, several sentences with new proper citations have been added firstly explaining which are the main factors affecting the proportionality of the CPUE (both for commercial and surveys data) and thus what the canonical standardization process consists of; secondly, specifying that such a standardization process has been incorporated with prediction grids accounting for the significant effects of independent (explanatory) variables on CPUE.

In other words, GAMs built on the basis of point values collected within the time series in variable conditions (e.g. different time, season / week of the year, sampling station, depth, associated bottom environmental parameter, etc.) can be used to produce standardized indices (as average values per year and season/survey); the obtained standardized indices refer to fixed conditions (e.g. a specific time of day) and, through the use of informed prediction grids (according to the spatial and temporal domain of data, each cell of the grid contains reference values for each covariate retained in the final model used to predict standardized CPUE), the influence of covariates and factors that vary in space and time (e.g. local depth, environmental or management conditions) was also taken into account.

The materials and methods section lacks of many useful details about how the analysis was conducted, requiring a substantial revision of this section of the manuscript.

Materials and methods were revised.

Data were not completely described and a vague idea of the spatial and temporal coverage (not supported by an adequate mapping support) was provided.

In Materials and method some more information about the design of both surveys were added specifying the spatial distribution of planned hauls according to the areas with different management levels. As suggested by reviewer#1, the number of filtered hauls of both surveys were also specified. Finally, in the supplementary material (Supplementary Figures S1-S4), annual bubble plots for each survey were added for both the standardized indices.

The analysis do not support the aim of the work. Indeed, even if the authors express the intention of “investigate how the environmental variables and the different management strategies can influence abundance and distribution of Nephrops”, the real effect of the environmental variables was not effectively tested. In fact, the analysis lacks of either a comparison between models using environmental variables and models not including those variables, or the estimation of the effective contribution of each variable to the total explained deviance, to highlight the real influence of those variables.

All the analyses previously presented were revised in order to better investigate the real effect of environmental and fishery management covariates. Three different models, both for density and biomass CPUE, were then built and tested: (i) a model involving only the main effects for all the covariates (i.e. mod INITIAL), (ii) a model without the environmental covariates (but with fishery management covariates; mod NOE), (iii) and a model without fishery management covariates (but with the environmental parameters; mod NOM). A parametric one-way ANOVA with post-hoc Tukey test has been conducted between these models and the AIC values calculated in the 10k-cross validation (repeated 10 times). In such a way it could be investigated if there’s a significant difference among those 3 models. The results confirm that the initial model, which include both environmental and fishery covariates, was significantly different from the others. By the comparison of mean AIC value calculated from the cross validation, it could be also proved that the initial model showed the best performance metrics (lowest mean AIC value); such result is in accord with the model presented in the last submission.

Furthermore, the final selected models include some variables arbitrarily retained even they were estimated as not significant. This is the case of some environmental variables but also of the “week” effect, used to standardize the CPUEs.

The final model presented in the last submission was revised accounting for the only significant terms. Hence the final model equation was changed (“week” and “BT” terms were excluded) and the related predictions were updated. The results are mostly in accord with the previous ones.

Notwithstanding, such standardization of the two seasonal surveys is also deeply impacted by the limited number of available data (due to the data filtration) and the high number of variables used to over-parametrize the models.

In this submitted version of the manuscript, the final model used to predict the standardized CPUE includes a lower number of explanatory variables reducing the possibility of over-parameterization. Nevertheless, in the discussions, the possibility that the low number of observations may negatively influence the predictive power of the model (which is however evaluated by RMSE) were stated.

Discussion section, even if it was well structured, needs to be adapted at the light of the revision applied to the other sections of the manuscript.

The discussion section was revised to better argue the model building process, the influence of environmental variables in the standardization modelling approach and their contribute to the predictions of catch rates also in relation to the related environmental maps.

In conclusion, I recommend to the Editors to reject the publication the manuscript in this version, anyway I suggest to keep open the door for a new submission, in case the authors will solve the following specific comments.

Line 32-33. The authors write “more accurate estimates of both biomass and density indices” but it is not clear in comparison with what they think it would be true. Please be clearer on this.
The estimated indices through GAM standardization should be more accurate than raw ones since modelling the effect of independent variables on spatial distribution and abundances removes most of the variability, as described in Maunder & Punt (2004). To achieve such a target , informed grids have been built for each year/survey combination and used to predict standardized CPUE; some sentences have been added in the abstract and in the introduction with the appropriate quotations to better explain the advantages of such an approach.

Maunder, M. N. and Punt, A. E. 2004. Standardizing catch and effort data: a review of recent approaches. Fisheries Research, 70: 141–159..

Line 42. I suggest to replace wording “model” with “modelling approach”

Changed

Lines 65-67. Gaps in space and time are not the only issues related to the “need” of standardize surveys CPUE time series. The authors should even take in mind eventual discontinuities in the protocol, in the vessels or gears used, as well as in the sampling intensity along the time series

A sentence has been added

Lines 67-69. Would the authors state that the CPUE standardization should be used to eliminate the effects excluding abundance and survey operational shortcomings? The sentence is not clear. Please, rephrase in a clearer way.

Rephrased also according to reviewer#1 suggestion.

Lines 69-71. In my opinion it is not clearly explained how standardization is able to produce more accurate estimates of both abundance indices and of related standard errors in a standardized time series in comparison to the raw data. I mean that it is important to better explain that the standardization process is driven by the prediction of the models in reference conditions (standard) such as for example a fixed reference grid or a fixed number of sampling points as well as a reference time period during the year (and the related environmental condition in the survey area).

A couple of sentences have been added explaining what the canonical standardization process consists of and why it leads to more reliable abundance estimates. In addition, the following paragraph was adjusted specifying that such a standardization process has been incorporated with prediction grids accounting for the significant effects of explanatory variables. Each cell of the grid thus includes reference values for each covariate retained in the final model used to predict standardized CPUE. A similar approach to model the spatio-temporal distribution and population abundance of a crustacean species has been quoted as example (Rubec at al., 2016)

Rubec, P. J., Kiltie, R., Leone, E., Flamm, R. O., McEachron, L., & Santi, C. (2016). Using delta-generalized additive models to predict spatial distributions and population abundance of juvenile pink shrimp in Tampa Bay, Florida. Marine and coastal fisheries, 8(1), 232-243.
Lines 111-113. May be the authors were speaking about standardization models otherwise I can’t understand why they are referring to population dynamic models

The sentence has been rephrased and accompanied by a new one explaining why the emergence behaviour of *Nephrops* should be considered in the standardization process.

Lines 116-119. Did the authors take in consideration the presence of distinct subpopulations in the Adriatic Sea? If not it is not clear to me how it is pertinent for the subject of the paper.

The presence of different subpopulations in the Adriatic Sea was introduced just to explain the importance of that specific and particular subpopulation that inhabits the Pomo/jabuka Pits. In this way the reader should better understand why more accurate information and tools are needed to support the assessment (as stated in STECF, 2016) and ecosystem models of *Nephrops* for the Pomo/Jabuka Pits area (Angelini et al., 2016)

Scientific T and EC for F (STECF). Mediterranean assessments part 2 (STECF-16-08). 2016. 2016. doi:https://doi.org/10.2760/015005

Angelini S, Hillary R, Morello EB, Plagányi ÉE, Martinelli M, Manfredi C, et al. An Ecosystem Model of Intermediate Complexity to test management options for fisheries: A case study. Ecol Modell. 2016;319: 218–232. doi:10.1016/j.ecolmodel.2015.07.031

Lines 146-154. Did the surveys share the same stations allocation in the common area or they have a different hauls allocation. The map do not allows to distinguish which is the position of the hauls of each survey. Please, add another map if needed.

Line 180. Details about the number of stations surveyed each year should be added. Few information about sampling intensity were provided.

In response to both comments, the 2 seasonal surveys share the survey design in the common area (i.e. western side); some additional hauls have been added in both survey plans since 2016. Bubble plots for each survey have been added in the supplementary materials (Supplementary Figures S1-S4) to better describe the distribution of the available data (CPUEs derived from hauls actually conducted in each survey).

Lines 204-206. Please, explain better how those data were used, in particular to assess the environmental variability over time. More details should be provided.

The previous paragraph and the first line of this one has been adjusted explaining that for each sampling station it was possible to associate the relevant environmental data aiming to model their relationship with the catch rate.

Lines 208-209. Not clear. Please, rephrase.

Rephrased and better argued

Line 224. Are those values the only outliers observed in both survey? Please specify it. Are those values from the same haul?

Also according to Reviewer#1 the “outliers” sentence was rephrased specifying that only the highest values were shown. They don’t come from the same haul.

Line 225. Did the authors try to use any other available distribution to test the model responses? Did the authors limited their analysis only to the Gamma based on any reference indication?

It is not specified in the manuscript, but numerous initial tests had been made on transformed and no-trasformed Gaussian distributions. All of these resulted in poor model performance metrics compared to models with Gamma distribution. A sentence describing why the Gamma family is ideal for the data distribution presented here has been added.

Lines 232-233. Wouldn't it be better to use the month or the quarter information to discriminate the seasons?

Even in this case many trials were done using the season (i.e. spring/autumn), the month when the survey was conducted for each year, and finally the day of each haul. The preliminary results had shown that the temporal scale described by the week of the year slightly increased the significance of such a term in comparison to the others. A sentence specifying these not presented results has been added.

Lines 235-237. Are the hauls of both surveys equally allocated between fishery allowed (Y), limited fishery (L) and fishery ban (N) areas.

The newly added bubble plots can probably directly answer this question. However, the planned hauls were equally allocated among areas with different management levels. Unfortunately, not all the planned hauls could be performed every year; on the other hand for each modelled year, except for 2018, at least one haul for each area was carried out.

Line 253. Why the authors did not use another type of smoothing factor for “week” ’s variable? May be a circular spline would be a good solution with such a variable.

According also to the reviewer#1’s comments, the circular spline was entered in the model only for the “week” term.

Line 255. How the maximum k parameter of 6 was fixed?

The number of basis functions (k) was fixed at 6 because the model does not support more knots due to the low number of data. To get to such a number of knots we initially started from 10 (default k of “mgcv” package in R) and then we descended to a k-value suitable for the cross validation.
The sentence specifying these not presented results has been adjusted.

Lines 259-262. The use of such a cross validation method is widely adopted but I think that such a low number of data (e.g. 1/5 of all the hauls) using a so high number of variables (e.g. 9 variable for the full model) could generate over parametrized models.

In order to reduce the possibility to generate overparameterization in the developed models the 5k-cross validation (repeated 20 times) was replaced with a 10k-fold cross validation repeated 10 times.

Lines 262-265. It sounds so strange to me such an arbitrary choice of retaining not significant variables in a model. The fact that those environmental variables are reported in literature to have a consistent effect on the Nephrops life and behaviours is the reason why their effect on the model response should be reasonably assessed and tested, provided that a significant response is observed in the given condition. It is also possible that the sampling design together with the limited number of data is not enough informative to the model about the effects of the environmental variables.

Following this comment, a parametric one-way ANOVA with post-hoc Tukey test has been developed between tested models and the AIC values calculated in the 10k-cross validation (repeated 10 times). The aim was to show if there’s a significant difference between the model involving all the available covariates and, on the one hand, that including the effect of fishery management actions (and not the environmental parameters) and, on the other hand, another including only the effect of environmental variables (and not the management actions). The results show that there’s a significant difference among those 3 models with the lowest AIC recorded for the initial one. Moreover the significance of each term of the initial model has been verified through a comparison among the 100 p-values obtained from the cross-validation leading to the exclusion of “week” and “BT” terms from the final model equation used to predict the standardized indices.

Lines 267-270. On the base of the consideration provided for lines 259-262, the selection of the best model based on such over parametrized models could be not really effective and informative.

The number of variables of the final model has been reduced from 9 terms to 7, in addition the model retains only the significant covariates verified through an evaluation of the number of significant p-values obtained from the cross-validation (as a rule of thumb if in 80% of cases the p-values was above 0.05, thus the covariate was included in the final model). Such a combination should reduce the possibility to generate over parametrized models.

Lises 285-290. It is very difficult to follow the global method of the paper giving the lack of many details along all the Materials and Methods sections. Provided that the predictive grids were derived from kriging analysis based on the data collected during the survey, how it was possible to produce predictive layers of each variable for each year when the survey were conducted in different weeks of the same season (18° week for spring survey, 45° week for autumn survey)? I suppose that the week was not the same along the time series otherwise it would no possible to use this variable in the models, but no details were provided. The way the authors used the predictive grids for the standardization process is really smocky and unclear. Further details should be provided about the spatial and temporal distribution of the data along the time series the better understand how the method was applied and assess if it is really effective.

The predictive layers of each variable for each year generated from kriging analysis were used as a proxy for the entire season because the environmental variables were generally collected in the same season in which the surveys were carried out. In such a way it could be modelled the seasonal variability on Nephrops’ CPUE by predicting an index for each season, accounting for both the effects of environmental parameters and fishery management measures in place. The M&M have been revised and more details about the model building, model selection and standardization process are now provided. In this new submission, the “week” term has been excluded from the final model equation used to predict the standardized time series. Moreover, with the aim to be more clear in the spatial and temporal distribution of the data, a bubble plot by year for each survey and CPUE has been added in the supplementary material.

Lines 298-300. How the mean CPUE indices were statistically compared? Please, provide some details about the method.

The CPUE indices were not statistically compared, the RMSE across the 10-fold cross validation repeated 10 times has been used to evaluate the performance of the prediction. In Figure 8 the mean raw CPUE (average of hauls values) were compared to the average of the standardized values obtained for each cell of the grid; this allowed to visualize whether the predictions were within the interquartile range (i.e. middle 50% of the observed data).

Line 318-321. It is not clear how confidence interval were derived for both explained deviance and AIC values for the final model. It seems that the authors reported the values derived from the models built on the 100 runs of 5-fold cross validation. If it is true, the author should report the values relative to the final model used to predict the CPUE indices.
The performance metrics (percentage of explained deviance and RMSE) relative to the final model have been now reported in the results section.

Line 323. What test was used to test the homogeneity of variance. No details were provided.

The Levene's test has been developed to test the homogeneity of variance. More details have been provided.

Lines 328-342. It seems the author used the same models to standardize two different surveys with different spatial and temporal allocations. In Materials & Methods section it is not clearly stated how many models would be built. As previously stated elsewhere in this review, the manuscript lacks of many useful details. Furthermore, being the week variable not significant in both the final models how can be this variable effectively used to standardize CPUE data, especially considering that the two surveys were conducted in two different seasons?

In M&M now has been specified how many models were built and the related equations with significance of each term and model performance metrics included within the text and in supplementary tables S5 and S6. The “week” covariate has been excluded from the final model used to predict the standardized indices.

Lines 409-411. If I am not wrong this discrepancy is correspondent with the year for which environmental data were not available. I suggest the author to consider the use of Copernicus environmental data, available with a resolution comparable with the standardization grids described in the manuscript and with a temporal resolution at the day and month (generally available since 1999).

The major discrepancy is actually related to the year in which the oxygen saturation value was not available and values from 2013 were used as a proxy; however as further discussed in the new version of the manuscript, one of the variables that showed a relevant impact on the predictions is the salinity. Salinity actually recorded in 2012 showed very low values if compared with the following years/season combinations (see supplementary Figures S5 and S6). These low salinity values were in correspondence with the maximum difference between observed and predicted mean values shown in 2012 suggesting that such a discrepancy in the prediction could be mainly due to these low salinity concentrations. However, the inclusion of other data sources is referred to as a potential future improvement of the model in the new version of the manuscript. Furthermore, in this case study the idea was to use data collected directly on the seabed simultaneously to the experimental trawl hauls, thus it was decided to test the above described proxy instead of including data from different sources, also trying to avoid possible bias due to different estimation procedures.

Lines 428-430. It would be useful to compare the results of a spatial analysis should with the observed data on the same geographic resolution. At least a bubble plot of both abundance and biomass by hauls of the two surveys should be provided to allow an effective assessment of the spatial results of the models.

Bubble plots for non standardized CPUEs (i.e. abundance and density) by year and survey have been added in the supplementary materials (Supplementary Figures S1-S4).

Line 433, 441. What does it mean: “The latitude is North, the longitude is East.”? Please better explain.

Fixed in the captions.

Lines 499-503. I agree with the author about the limited amount of information available for affectively modelling a so complex phenomena. In my opinion, the authors would like to use a huge amount of variables in comparison with the available amount of data. In such an analytical condition, the models may not be really informed about the real system variability along all the available covariates. Furthermore, retaining useless variables and not significant effects could only generate bias in the model estimations. The analysis lacks also in the real possibility to assess the real importance and effects of the environmental variables. In the manuscript it is not reported at all which is the difference between models with and without the inclusion of environmental variables, even if at lines 134-135 it is stated that the purpose of the study is to “investigate how the environmental variables and the different management strategies can influence abundance and distribution of Nephrops”. The authors can further consider to estimate the effective contribution of each variable to the total explained deviance to highlight the real influence of the environmental variables.

Following this comment some relevant methodological changes have been applied to the model selection. The number of variables included in the model and used to predict CPUE has been reduced, retaining in the equations only the significant terms; this should improve the reliability of the predictions avoiding the generation of biases in the model estimation. With the aim to assess the real contribution on response variables and the effects of the environmental variables as well as fishery management actions over years, a parametric one-way ANOVA with post-hoc Tuckey’ test has been developed. Such statistical analysis have been conducted among a full or initial model including all the available covariates, a model with environmental parameters (and not the fishery management factors), and another one without environmental parameters (but with fishery management factors). The results showed a significant difference among these models. Finally, with the aim to detect which of those models was statistically better, a comparison by considering the mean and the standard deviation of the AIC values obtained from the cross-validation has been developed. The results showed that the models with environmental parameters perform better than the ones without them. The model used to predict the standardized CPUE (i.e. final model) has been evaluated through the RMSE tuning it on all the data at hand once the significance of each term was verified.
