# Supplementary material for: Accounting for environmental and fishery management factors when standardizing CPUE data from a scientific survey: A case study for Nephrops norvegicus in the Pomo Pits area (Central Adriatic Sea)
Source: PLoS One. 2022 Jul 14;17(7):e0270703. doi: 10.1371/journal.pone.0270703 (PMC9282463; doi:10.1371/journal.pone.0270703)
Supplement: Attachment — Submitted filename: Chiarini et al. (PlosONE)_Response to Reviewers.docx [file pone.0270703.s003.docx]

**Review Comments to the Author:**

Reviewer #1: This manuscript presents GAM-based models of Nephrops survey data, identifying variables associated with areal pattern in density and/or biomass. Some details of the methods are confusing or not explained. Importantly, the concept of standardization is not well thought through and could be better explained. This might also be achieved with a clearer example(s) of how unstandardized data are used currently and what quantitative difference is made by using a multiple variable model.

The positioning of the analysis as ‘standardization’ is possibly appropriate, but not carefully presented. Standardization might usually apply to a correction for some sort of sampling variable (e.g., different tow lengths standardized to a constant length). The authors present an analysis of factors effecting the response variables, but this does not seem to be a standardization. It would help to be more explicit and compare the fitted surface to a not-standardized equivalent. For example multiplying the average density or biomass by the total area of the fishery (= non-standardized for any covariates).

First of all, thank you for your comments and suggestions which are very appreciated. In the aim to clarify what is intended for CPUE standardization within this study and based on definitions given by Maunder & Punt (2004), Maunder et al. (2006) and Forrestal et al. (2019), the introduction section has been modified.

- Maunder MN, Punt AE. Standardizing catch and effort data: A review of recent approaches. Fish Res. 2004;70: 141–159. doi:10.1016/j.fishres.2004.08.002
- Maunder, M. N., Sibert, J. R. Fonteneau, A., Hampton, J., Kleiber, P., and Harley, S. J. Interpreting catch per unit eﬀort data to assess the status of individual stocks and communities. ICES Journal of Marine Science. 2006; 63: 1373-1385
- Forrestal, F. C., Schirripa, M., Goodyear, C. P., Arrizabalaga, H., Babcock, E. A., Coelho, R., ... & Walter, J. Testing robustness of CPUE standardization and inclusion of environmental variables with simulated longline catch datasets. Fish. Res. 2019; 210: 1-13.

While it may help to standardize variation in space (by providing more data), the use of ‘year’ is problematic as part of a standardization. The point of CPUE is often to follow changes over time (in the fishery of interest). The changes with year identified in the analysis presented in the manuscript are interesting, but the phrase ‘standardizing by year’ doesn’t make sense in the conventional use of CPUE data. This is also reflected by other comments highlighting the confusion between abundance and catchability. The revision does not do a good job of considering the wisdom of mixing year (abundance trend) and time of day (catchability change) in the analysis.

Thanks for the comments, we hope to have now better clarified in the text what is intended for abundance and catchability (see previous reply); unfortunately we were not able to identify where in the text the sentence “...standardizing by year…” was found by the reviewer, however as indicated in the “Model building section” (within the Materials and Methods chapter), the temporal covariate year (“Yr”) was directly included in the models as an interaction with the factor “Fishery” (using the “by” argument in the smooth functions) in order to model potentially different trends over years for each of the different fishery management levels (that changed over time and space).

The methods are unclear is several respects:
1) The datasets were merged (line 233). How were they merged? If they were merged, why are there two response variables (density and biomass).
As already explained in the “Data collection” section (within the Materials and Methods chapter), the original dataset used within this study consists in catch data (both in terms of biomass and number of individuals per haul) derived by experimental trawl hauls carried out in the study area during two annual surveys (UWTV survey carried out in spring and ScamPo survey carried out in autumn). The catch data allowed to obtain for each performed haul CPUE as biomass and density indices (kg/km^2^ and N/km^2^ respectively).

To make it more clear, now the text has been modified adding the sentence *“The ''UWTV'' (hereafter referred to as spring) survey principally aims to quantify Nephrops burrows through video analysis of seabed footage [85,86] but during this also experimental trawling activities are carried out [63,72,87]; these latter data on trawling were those considered in the context of this study. Instead, the ''ScamPo'' (hereafter referred to as autumn) survey is mainly meant to monitor the effects of the management measures on commercially relevant demersal species (including Nephrops) [72].”*

and throughout the text now it is referred to the two time series as *spring* and *autumn*.

In the model building process the seasonal datasets were merged with the aim to obtain larger and thus more robust datasets to train the GAMs.

Accordingly two final GAMs were obtained (one for biomass and one for density indices). Then the availability of 2 seasonal grids including all environmental parameters and covariates used to build the models allowed to obtain predictions for both indices and both seasons.

2) Analyses of the UWTV data have a factor for time of day. The text implies that trawl protocols were the same (line 214), so that videos were taken at sunset and sunrise. However, videos count burrows. How can the density of structures like burrows logically vary between sunrise and sunset (Table 2)? This does not make sense.

As for the previous comment, probably there was a misunderstanding, in fact, as explained in the “Data collection” section (within the Materials and Methods chapter), although the “UWTV” survey principally aims at collecting seabed footage, in this paper only the catch data from the additional trawling activities were considered. We hope that this has been now better clarified in the text (see previous reply). Furthermore, trawl hauls in both considered surveys (spring and autumn) are carried out at sunrise and sunset, therefore, also according to available literature (e.g Aguzzi & Sardà, 2008) we considered reasonable investigating possible differences related to the factor for time of day.

- Aguzzi J, Sardà F. A history of recent advancements on Nephrops norvegicus behavioral and physiological rhythms. Rev Fish Biol Fish. 2008;18: 235–248. doi:10.1007/s11160-007-9071-9

3) The partial effects plots are very similar (figure 2 and 3). If the data were merged as stated, why are there separate plots? If the data were not merged, why are the plots so similar?
As for comments 1 and 2 the interpretation of figure 2 and 3 has probably been influenced by the misunderstanding that we hope to have clarified in previous replies and related changes in the text.

Indeed the spring and autumn catch datasets were merged as explained in the “Model building” and “Model selection” sections (within the Materials and Methods chapter) with the aim to obtain two final GAMs, one for each response variable (biomass and density indices), resulting in two different plots of partial effects. The reason behind the similarity between these two plots is attributable to the resulting significant terms in the equation of the final models (eq.5) for both indices (i.e. biomass and density indices; see Table 1).

4) The same point apples to figures 4 – 7. It is not clear what data were used to fit the models. They look very similar plots. Is this (for example) extrapolation of a spring model with autumn data or snapshots of a general model produced from all data? Why do we need 4 plots – this point should be made clear or fewer plots presented (but again, the sources of data need to be clear).

As for the previous replies we hope to have now better clarified the data sources. The overarching goal was to obtain a model for biomass index and one for density index (see also reply to comment 1), then relying on the availability of environmental data for 2 different seasons, separate informed grids for each year/season combination were built and used to carry out predictions. These lead to obtaining 4 plots: spring and autumn for biomass index predictions and spring and autumn for density index predictions. Furthermore, also accordingly to review #3 comments, a paragraph clarifying why all the analyses are repeated for both density index (N/km2) and biomass index (kg/km2) has been added at the start of the Discussion chapter (at lines 643-649).

5) The conversion from burrows to numbers per km2 does not appear to be explained. Burrows are not subject to debates about catchability, although the (unstated) conversion to density will have some assumptions.
In this study only the catch data from trawling activities carried out during the surveys were considered, no density indices derived from burrows counting were taken into account.

6) The models are fit with “gamma distribution with logarithmic link function” (line 251), but this is contradicted by “logit link function” on line 301.
Thanks for the comment, the models are fit with gamma distribution and logit link function. Lines 277-281 were modified as follows:

*“Therefore, in the GAMs, a Gamma distribution with logit link function was assumed for both response variables; this distribution is one of the most used for environmental analysis [93], works well for positive-skewed data [94] and, owing to the logit link function, makes the model additive and therefore easier to interpret.”*

7) Symmetric standard errors are not appropriate with skewed data (e.g., figure 8)
According to Wood (2011), the symmetric standard errors are appropriate as they are the standard errors of the mean sampling distribution which is known to be gaussian independently of the distribution the sample comes from.

- Wood SN. Fast stable restricted maximum likelihood and marginal likelihood estimation of semiparametric generalized linear models. J R Stat Soc Ser B Stat Methodol. 2011;73: 3–36. doi:10.1111/j.1467-9868.2010.00749.x

8) R2 is (line 433) is not a good measure for model fit with skewed data. R2 is also not a good measure of model performance in this sort of exploratory multiple variable setting. The average r2 (if it can be calculated given the skewed data) from cross validations is a better measure of model fit.
The adjusted R^2^ reported in the paper is the value of the final tuned model fitted on the whole dataset, as such it is a single value and it is reported just to give an idea on how good the final tuned model fits the data. As stated in Wood (2017), the adjusted R^2^ is a proper parameter to evaluate the model performance. In our paper, the model performance, during cross validation, was assessed by evaluating the root-mean-square-error (RMSE, line 425) and the adjusted R^2^ was added just for information.

- Wood SN. Generalized additive models: An introduction with R, second edition. Generalized Additive Models: An Introduction with R, Second Edition. CRC Press; 2017. doi:10.1201/9781315370279

9) Why are observations missing from the partial plots for year in Figures 2 and 3?

Thanks for the comment, it was due to an error in the script used and now these observations have been added in the partial plots for year.

Reviewer #2: Chiarini et al. analysed data from two seasonal surveys for addressing the interesting aim to investigate how some environmental variables and different management strategies can influence abundance and distribution of Nephrops norvegicus and to produce standardized CPUEs. The dataset comes from two surveys performed in an area, the Pomo/Jabuka Pits, subjected to different management measures in time and space. The authors used GAM models and found that bathymetry, fishing pressure, dissolved oxygen and salinity proved to be significant drivers influencing catch distribution for N. norvegicus in the area.
The new manuscript is largely well written and easy to follow, and I appreciated the authors' effort to address all the raised questions and concerns from the previous referees.
Used methodology appears clearly explained and their choice appropriate for the analysis done.
The final discussion chapter appears exhaustive, with a good selection of reference literature.
I only have some very few minor questions/comments listed below.

Figure/table captions: please check all captions and relative citation in the text according to the formatting guidelines. (i.e. Cite figures as “Fig 1”, “Fig 2”, use bold type for the figure titles, etc.)
Thanks for the comment, all the Figures/Tables captions and citations along the manuscript as well as within the supporting information were modified according to the formatting guidelines.
Line 213. It is not clear: does “However, the planned hauls were equally allocated among areas…” refer to the performed hauls in the years when not all hauls could be done? If it is so, please change it with “However, the remaining performed hauls were equally allocated among areas…”. Instead, if the sentence refers to all the planned hauls in general, indicating that the management level is somehow taken into consideration in the planning of the two surveys, delete “however”.
Thanks for the comment, this sentence referred to the performed hauls in the years when not all the planned hauls could be carried out. The sentence has now been changed in accordance with this indication.
Line 291. “for both for density and biomass CPUE” change in “for both density and biomass CPUE”
Thanks for the comment, the sentence has now been accordingly changed.
Line 398 “The Tukey post hoc test evidenced that “modINITIAL” was more statistically sound (lower AIC values) than “modNOE” and “modNOM””. It is not well clear why the Tukey test showed that the modINITIAL resulted more statistically sound: the test performs a pairwise comparison and showed a significant difference among all the pairs of models. The choose of the best model seems to be done by the lowest mean AIC values from the cross validation (Table S5).
Thanks for the hint, in the aim to be more clear the sentence was changed as follows:

*“The Tukey post hoc test on the mean AIC showed significant differences for all the compared pairs of models both for biomass and density indices (S3 and S4 Tables). “modINITIAL” has the statistically lowest average AIC value than “modNOE”, “modNOM” and “modNOEM” (respectively not including environmental parameters, fishery management factors and both; S5 Table); “modINITIAL” may thus be considered the best model.”*

Line 431. The tables should be tables 2 and 3 (not 1 and 2)
Changed
Line 438. The tables should be tables 2 and 3
Changed
Line 440 Table 2
Changed
Line 447 Table 3
Changed
Line 457-462. The sentence is too long, please, divide it.
The sentence was divided
Line 462. These should be table 2 and 3
Changed
Line 489 “after which level it...” change in “after which it…”
“*Level*” was cancelled
Line 541 Correct the figure caption with “…The "UWTV" time series is on the left (a and c panels), the "ScamPo" time series is on the right” (b and d panels)
Caption adjusted
Line 550 “…increment of about 67%”: please verify the percentage, from 24.9 to 37.33 it seems more an increase of about 50%

Percentage corrected

Reviewer #3: The study by Chiarini et al., presents an analysis to standardize N. norvegicus CPUE from 2 surveys from 2012 to 2019 using GAM models. In particular the authors i) investigate how the environmental variables and the different management strategies can influence abundance and distribution of Nephrops, ii) produce standardized CPUEs, to be used as reliable inputs for population dynamic and ecosystem models. The data are coming from two seasonal surveys, the areas associated with each survey being under different management measures.
I found the objectives of the study very interesting, but I have major comments about how the method and the results can addressed the main objectives of this paper. I then recommend major revision and would be very interested in seeing their responses to my questions/comments.

Major comments

Major Comments 1 :It was very difficult to me to figure out the spatiotemporal dimensions of the data and the model :

Major Comments 1.a :Data: it was difficult to me to understand the resolution of the observed data :
- Is there a sampling grid ? are the bubble points in Figure S1-S4 at the same locations than the green triangles in Figure 1 ?
Thanks for the comment. The position of the planned hauls originates from an originally random stratified design according to bathymetry and legislative or operational limitations (sentence added in the text at lines 228-230).

Fig 1 shows the location of all the planned hauls (the same for both the surveys according to the survey spatial domain). Yes, the green triangles in Fig 1 correspond to the hauls presented in the S2-S5 Figs (reference to these figures has been added in the text at line 231); S2-S5 Figs show the hauls actually performed in each year/survey.

- When I look at figure S1-S4 : are there the raw observed data ? Depending on years some hauls seem to have zero densities (example in FigS1 there in 2017 in the northwest there are positive catches but then at the same point there is not catches). So, this makes me think that some observations (CPUE) are equal to zero. I am then surprised that the models do not account for catches=0 (by using a zero inflated approaches or using a negative binomial distribution). Especially, there is no sentences or explanations about how the model deal with zero (the percentage of zero for each year and each survey ?)
Thanks for the comment. S1-S4 Figs show the normalized observed data. Following this comment, these figures have now been modified in order to account for 0 values (catches = 0) by indicating the “null” hauls with a cross. As can be easily seen, the figures show zero values only for one haul in spring (the same in 2 different years 2016 and 2017). These 2 hauls were however removed from the dataset because they did not include all the information to be tested in the model i.e. CTD information (lines 262-266); this information has now been included in the text in the “Model building” section (within the Materials and Methods chapter) at line 269. The use of zero inflated approaches was therefore considered superfluous in this specific case study, hence a Gamma distribution with logit link function was settled because it works well for positive-skewed data (line 280).

Major Comments 1.b :Model: I find very interesting to account for different levels of time within the model: year/week/date of day. However, it is difficult to me to understand the dimensions (i.e time and space) of the model. If the model accounts for both weeks and years, it is complicated to me to understand the ecological meaning of the effects of bottom temperature (same for oxy, Sal). According to my understanding, the model (eq1) estimates here an average effect of temperature (and oxy, Sal) on CPUE. But because the authors are not providing any hypothesis about the mechanisms tested, I am a bit lost. The authors have data (and the model) to estimate the seasonal changes but here they use those data to estimate an average effect (which can be interpretate I guess as a niche effect/habitat suitability). So at the end of the day, I am curious to know why putting so much efforts in collecting so many great data (I am wondering if yearly averaged temperature (oxygen/Sal) outputs from oceanographic models would have provided the same kind of information for example?).

Thanks for this comment. Indeed the term week was tested in model (eq1) in order to account for possible seasonal effects (after testing also other temporal scales e.g. season, month; see line 306); unfortunately, following the cross validation approach, the week term resulted not to be significant (see Table 1 and lines 464-466), hence it was not included in the final model (eq. 5).

For what may concern environmental variables, for the model building we decided to use the available set of environmental parameters directly collected in proximity of each trawl haul (CTD casts performed in the close proximity of each trawl haul). Indeed, due to the oceanographic peculiarities of the study area, CTD (Conductivity Temperature Depth) casts were also carried out during the surveys in order to capture spatiotemporal variations of environmental parameters at small scales (sentence added in the text at lines 244-250). Therefore point estimates of directly collected bottom environmental data were associated to each CPUE value instead of averages from oceanographic models.

Through an interpolation method (see lines 416-419), the same available CTD datasets were also used to build 2 informed grids for each year accounting for the 2 sampling seasons (spring and autumn; S6-S11 Figs). These grids, accounting for temporal variability of bottom environmental parameters as well as changes in fishery management measures, were used to perform seasonal predictions for each year. We had the chance to rely on available dataset that we considered more reliable than those that can be produced by oceanographic models. In addition, finding reliable data on bottom environmental variables from oceanographic models to such a small spatial scale (see study area) is generally a major challenge.

Again, I think that if the underlying mechanisms linking the covariates (in time (year, week, date of day) and space) and CPUE would have been better defined, I think it would have helped me a lot to better understand what is going on with the model.

In order to help the comprehension of the underlying mechanisms linking the covariates to CPUE we modified the text of the section “Model Building” (within the Materials and Methods chapter, see lines 285-310). For example, for what may concern the ToD covariate, we tried to better explain the basic assumption on which we rely; previous knowledge about the mechanism linking the temporal variations in catch rate for *Nephrops* was also stated in the introduction (see lines 129-138). Indeed, as suggested by Aguzzi & Sardà (2008), accounting for the availability of *Nephrops* to trawlers according to the time of the day and season (due to its circadian emergence rhythms) may lead to more reliable estimates of population abundance.

More in general the aim of the study is to find a functional relationship, if this exists, between CPUE and some covariates representing the effect of time (year, week, time of the day), space (latitude, longitude), environment (depth, temperature, salinity, dissolved oxygen) and fishery management (fishery allowed, limited or banned). This functional relationship will be pursued by fitting a GAM model on the data. In case of a good fitting, the model could be then used both for CPUE forecasting and to assess which covariates, among those considered, mostly influence the CPUE.

- Aguzzi J, Sardà F. A history of recent advancements on Nephrops norvegicus behavioral and physiological rhythms. Rev Fish Biol Fish. 2008;18: 235–248. doi:10.1007/s11160-007-9071-9

Major Comments 1.c :Covariates: Which covariates are varying in space and time ? I found figure S5 and S10 very useful to understand the spatiotemporal variation in environmental covariates. However, it is difficult to me to picture exactly how vary the fishery covariates. My understanding is that they are spatially defined (Zone A, B, C) and temporally defined? Can you please provide a table or a plot of those fishery covariates? It will help the reader to better understand how those covariates vary in time and space.
Thanks for the precious hint. The fishery covariates are definitely spatially and temporally defined. To make it clearer Fig 1 was modified and a new figure specifically showing changes in space and time of the fishery management measures was added in the supporting information file (S1 Fig); following what we already did for environmental covariates, now Fig S1 provides different plots for each of the fishery restriction implemented in the FRA of Pomo/Jabuka Pits over years.

Major Comments 2 : Because one of the objective of the paper is “to produce standardized CPUE to be used […] for population dynamic (line 148-150), I would have expected more information about the current population dynamic used for this species : actually is there one ? if yes why does it need new standardized CPUE ? how different are the new standardized CPUE produced in this study with the current CPUE used?
Because now the standardized CPUE are defined for each season, I am wondering if they will be able to be implemented in the population dynamic model, is this model seasonally defined ?
Regarding the objectives of the paper “to produce standardized CPUE to be used […] for population dynamic (line 148-150)” , I think we are missing some context/background about how standardized CPUE are/could be used in the population dynamics of this species
Recent population dynamic models for *Nephrops* in the Adriatic Sea were provided by STECF.In this stock assessment the fishery-independent data were from the MEDiterranean International Trawl Survey (MEDITS; Bertrand et al., 2002) which is the main index of abundance available for Mediterranean demersal resources. However, as stated in Carpi et al. (2017), MEDITS data suffer general issues about the inefficiency to catch *Nephrops* (due to its burrowing behaviour) as well as inadequate spatio-temporal specification (the survey does not follow the spatio-temporal protocol in all years) and highlighted the need for more accurate information and tools to support *Nephrops* management (see lines 146-152). In fact, these indices may not be representative of the real population because they are not dealing with the emergence rhythm of this species which alters its availability to the fishery thus influencing indices estimates (see Introduction chapter at lines 129-138 and “The importance of targeted sampling protocol for Nephrops” section within the Discussion chapter at lines 834-841). The raw CPUE time series treated in this work were never included in an official stock assessment before (see new lines 640-642in the Discussion chapter). As stated at lines 848-861, due to its intrinsic survey design, it would be relevant to include them in stock assessment models to overcome the issues related to surveys non specifically targeting this resource. Furthermore, the standardization process helps to overcome traditional survey issues such as the discontinuities in sampling intensity or strategy as well as the presence of gaps within the time series (see lines 77-80).

About the seasonal effects, the last population models used to assess *Nephrops* in the Adriatic Sea were not seasonally defined. However, in the near future stock assessment models such as the one developed by I.Tuck at el. for similar species (i.e. *Metanephrops challengeri*) (2017) that need to be fed by seasonally defined indices, could be implemented. A paragraph clarifying the importance of adopting seasonal indices within a *Nephrops* stock assessment has been added in the section “Spatio-temporal distribution of estimated CPUE” (within the Discussion chapter) at lines 679-694.

- Aguzzi J, Sardà F, Allué R. Seasonal dynamics in Nephrops norvegicus (Decapoda: Nephropidae) catches off the Catalan coasts (Western Mediterranean). Fish Res. 2004; 69: 293–300.
- Tuck, I. D. Characterisation and length-based population model for scampi (Metanephrops challengeri) at the Auckland Islands (SCI 6A). New Zealand Fisheries Assessment Report, 56. 2017.
- Bertrand JA, De Sola LG, Papaconstantinou C, Relini G, Souplet A , Gil de Sola L, et al. The general specifications of the MEDITS surveys. Sci Mar. 2002;66: 9–17. doi:10.3989/scimar.2002.66s29
- Carpi P, Scarcella G, Cardinale M. The Saga of the Management of Fisheries in the Adriatic Sea: History, Flaws, Difficulties, and Successes toward the Application of the Common Fisheries Policy in the Mediterranean. Front Mar Sci. 2017;4. doi:10.3389/fmars.2017.00423

Major Comments 3 :To account for spatial variability, I am curious to understand why the authors planned to account independently for latitude and longitude and not for the interaction which I guess could be a better proxy of the spatial location (Potts and Rose, 2018).
The reference was very appreciated. However, the Variance Inflation Factor (VIF) analysis showed that longitude covariates (X) had an extremely high multicollinearity value (lines 321-323); therefore, it was excluded from the model building process avoiding the inclusion of an interaction between geographic coordinates.

Major Comments 4 : I do not understand why all the analysis are repeated for both abundance density (N/km2) and biomass density (kg/km2) (the authors call it biomass it is biomass/area). All tables and figures are repeated without a clear message.
Thanks for the comment, at lines 240-243 now we tried to better specify how the two indices were calculated and how we refer to them through the text. The reason behind repeating the analysis for both available indices (i.e. N/km^2^ and kg/km^2^) is that we wanted to investigate possible differences between the trends of the two indices. Indeed, especially for this species (see Johnson et al. 2013 and new lines 643-646), the two indices do not always have similar trends as in this case; for example if the average size increases and the total number of individuals decreases (larger individuals at lower density index may result in constant biomass index).

Furthermore both the indices could be helpful to implement stock assessment models. Some stock assessment tools can be fed alternatively by both biomass and density indices (e.g CASAL, SS3). A paragraph clarifying why all the analyses are repeated for both density index (N/km2) and biomass index (kg/km2) has been added at the start of the Discussion chapter at lines 643-649.

- Johnson, M. P., Lordan, C., & Power, A. M.. Chapter two-habitat and ecology of nephrops norvegicus. Adv. in Mar. Bio. Academic Press, Cambridge. 2013. 27-63.

Major Comments 5: In the results section, it was difficult to me, to extract a clear message and arguments leading to meet the objectives of this study. For example, line 495-497 (also see line 531-534), this sentence presents results and explains how those results have been compiled (which according to me should be in the M&M). As a consequence, I think this makes the ‘results section’ a bit unclear. Also, it is really not clear to me (see comments 4) why there is so many comparisons and figures between densities and biomass. I do not understand how this help to meet the objectives of this paper.
Please see reply to comment 4.

Major Comments 6 : I think the discussion is missing a strong paragraph about the method used and other potential methods. Especially because the authors highlighted that GAMs are the best for this kind of study “in this study GAMs were selected as the best”. Which I think is not false but at least should be discussed ( it is more complicated than just saying GAMs are the best”)
According to this and other comments, the sentence at line 600 (new line 672) stating that GAMs are the best for this kind of study was modified with “*GAMs were selected as suitable tools*”. The choice for GAMs has now been better discussed in other sections of the manuscript (see Introduction chapter and “Filling the gaps of a time series through standardization with GAMs” section within the Discussion chapter).

In the introduction the authors highlight that considering for spatial autocorrelation is important (line 103). But in the method proposed in this study, they do not account for it (or maybe just through the smoother term for Latitude and I am OK with that). But I think it would be interesting to discuss this point in the discussion section. What information are we missing when not considering spatial autocorrelation, how can we account for this spatial autocorrelation better (Thorson, 2019; Brodie et al., 2020)
Thanks for the comment, since indeed we did not treat the spatial autocorrelation argument and overcome this by excluding correlated variables (please see reply to comment 3), we decided to modify the sentence at line 103 (within the Introduction chapter of the previous version).

“*Spatial autocorrelation*” was thus removed from the sentence (line 108) because the GAMs presented in this study do not account for it.

Also an important element should be discussed : what are the best models to answer those two questions : (I) investigate how the environmental variables and the different management strategies can influence abundance and distribution of Nephrops, (II) produce standardized CPUEs. One model can highly perform for question (I) and poorly perform for question II and inversely. Please see the very interesting discussion in (Brodie et al., 2020) “if model purpose is to best explain the environmental processes underlying species distributions, then BRTs and GAMs would be a best choice” , “model purpose may be to obtain robust abundance indices with associated standard errors, in which case VAST would be the best choice. In such cases, the most accurate abundance indices could be included in stock assessment”.
Thanks for the comment, this relevant information has now been added in the Introduction chapter (please see lines 110-115) and a better explanation on the choice of GAMs has now been added in the “Filling the gaps of a time series through standardization with GAMs” section within the Discussion chapter) at lines 672-677.

Intermediate/Minor comments

line 67: I do not know what “strategy changes” is in such a global context
Changed in “*management strategy changes*”
line 116 : what does “etc…” mean here ? I think it is important to define the main ecological process driving the population dynamic of Nephrops, so here “etc…” seems irrelevant to me.
The sentence was adjusted and “*etc..*” was removed (see lines 137-138)
Line 117-119 : Can we know more about the ecological mechanisms linking oxygen, salinity and bottom temperature to nephrod life cycle (or are they random correlations ?)
No, they are not random correlations. Indeed in order not to burden the introduction section, we preferred to give more references in the “Model building” section (within the Materials and Methods chapter) about all the environmental drivers entered in the model (oxygen, salinity and temperature), that are already known to influence *Nephrops* life cycle and emergence behaviour (see lines 122-128) and then address in detail within the discussion the ecological mechanisms linking these variables to the species life cycle and ecology (please see section “Roles of environmental variables in the standardization models”).

Line 127: by “differences over time”, do the authors mean, time of the day, and season and year ? It could help to better understand the temporal resolution of the study if the authors could be more precise
Thanks for the comment, referring to the citations reported in the prior lines, the sentence has been made more explicit (i.e. time of the day and season).

Line 222 -229 : Can the authors explicitly define the environmental data ? here I can only guess : temperature (bottom or surface ?) and oxygen (bottom or surface ?). What about salinity which is then use in the model.

Thanks for the comment, according to this and comments made by other reviewers this paragraph was revised (please see “Data collection” and “Model building” section within the Materials and Methods chapter).

Line 231 : what are the biotic and abiotic variables.
Thanks for the comment, the sentence has been modified in *“The effects of spatial and temporal variations of environmental parameters and fishery management measures on the CPUE indices were modeled by GAMs due to the high flexibility of the latter [31,91].”*

Line 250 : why a gamma and not a lognormal for example. I am not sure to understand the justification for a gamma distribution here.
In our dataset, no zeros are present (see reply to Major comment 1a), therefore we used a Gamma function which has, as support, the positive real axis + zero, while the lognormal has only the positive real axis without the zero.

Line 253 : I would say that the log-link function is mostly useful because the effects on log(CPUE) are then additive and it is therefore easier to understand/interpret
The sentence was changed according to this and other comments (please see lines 277-281)

Line 254-258 : Here again, I think it would give more power to the study if we could understand the underlying mechanisms linking CPUE and all covariates (Y, X, D, Yr, week, ToD, BT, Sal, Oxy, Fishery)
Thanks for the comment, following this and other comments, the importance and dimension of all covariates has now been better expressed in the text, please see lines 285-316

Line 260 : I do not know a lot about this fishery : are the restriction on fishery only occurring at the Year level or also at a seasonal level (for example the buffer zone). I am trying to understand here if the interaction between fishery and time should be only fishery*year or also fishery*weeks.
Maps about the different spatio-temporal fishery restrictions/bans implemented in the study area are now provided in the supporting information (S1 Fig). The fishery management measures occured at year level hence the interaction between fishery and time was managed to be only fishery*year.

Line 276: Why “few”? there are 3 covariates: oxygen, salinity and temperature. They represent an important amount of information, so I will not use the word “few”.
The sentence was changed removing the word “*few*”.

Line 286 : with which other covariates, longitude is correlated : I think it might be an interesting information to understand which covariates carry the same amount of information.
The longitude (X) is mainly correlated with the latitude (Y). This information has been added in the text at lines 322-323. Moreover the “X” term showed the highest VIF value (3.2526), while the “Y” term was the second highest value (3.1693); once “X” was removed, the VIF values for all the remaining covariates were below 3, and the VIF value for “Y” term substantially collapsed (1.3088).

Line 291 : At this point of the manuscript I do not understand why the authors are interested in both density and biomass. What are they investigating differently, when using biomass or density ?
Please see reply to Major comment 4, introduction and discussion chapters at lines 643-649.

Line 294 : It is difficult to me to understand the dimensions (spatial and temporal) of the variable CPUE. Here CPUE are defined for each latitude, longitude, week, years, others ? please define better CPUE, maybe add subscript for space and time ?
According to this and other comments we have tried to better clarify the dimension of CPUE at lines 240-243 in “Data collection” and “Model Building” sections (within the Materials and Methods chapter).

Line 300: is it a yearly, and/or spatial and/or weekly error term? Again, I would recommend to better defined the dimension of the variables in the model
The error term shown in all the equations is a general error term. The dimensions of each spatial covariate included in the model has now been better specified in the “Model building” section (within the Materials and Methods chapter, lines 287-288, 293, 304-305, 314-315).

Line 313 – 320 : How do we know that the terms Y, D, week and Yr that are common to equation (2) and equation (3), can explain variations in CPUE (are they significant ?).
So here a suggestion : I am wondering if first, it would not make more sense to figure out a model M0 with no environmental and fishery covariates, with only significant effects of covariates (Y, D week, ToD) on CPUE.
And then, once model M0 is validated, define model M2 (M0 + environmental covariates) et M3 (M0+fishery covariates) to figure out if environmental covariates (M2) explain more OR less variability than fishery covariates (M3). And finally, M4 will be M0 + Env.cov +fishery.cov (with only the covariates having a significant effects), which will be used to produce standardized CPUE.
As suggested, another model with no environmental and fishery covariates was added in the model selection process (named “modNOEM”). This section aimed to compare the obtained AIC values among all simplified models (originary only “modNOE” and “modNOM”, respectively not including environmental parameters and fishery management factors) and the initial one (“modINITIAL”, which includes all the available explanatory variables) through a first 10-k cross validation repeated 10 times; therefore, while adding another simplified model, all the subsequent outputs needed to be modified updating the model diagnostics values. In practice, the outputs from (1) Levene’s test for the homogeneity of the variance, (2) the one way anova with (3) Tukey post-hoc test for multiple comparison and (4) the main AIC values have now been all updated for each tested model. In the “Model selection” section (Materials & Methods chapter), the equation of “modNOEM” was added; within the results chapter of the “Model selection and fitting” section (lines 440-447) all the outputs from the statistical analyses were updated despite being extremely similar to the previous ones (no major changes that could affect discussions of previous findings were observed!). Therefore, the supporting information was as well accordingly changed to include the “new” results (S1-S5 Tables). The number of significant p-values obtained by the first cross validation analysis for all the covariates included in “modINITIAL” (Table 1) has not undergone any changes.

Also, if the authors want to answer the question they asked in Introduction: “investigate how the environmental variables and the different management strategies can influence abundance and distribution of Nephrops” I think they should be able to estimate the part of variance explained by each covariate, which will help them to conclude (i) about how the selected environmental influence abundance and (ii) discuss the underlying mechanisms.
Thanks for the comment, now an investigation on the deviance explained by each term of both final models was added, please see Materials and Methods (lines 395-397) and Results chapters (lines 485-488), and table S6 within the Supporting Information.

Line 330: I am not sur to know what is “the training data set”
The training dataset refers to the subset of data randomly extracted from the entire available dataset

on which the model is initially fitted and then applied to the test dataset for prediction and comparison (Hastie et al., 2017). Lines 371-376 have been now modified in the attempt to make it clear.

- Hastie, T., Tibshirani, R., Friedman, J. H., & Friedman, J. H. (2009). The elements of statistical learning: data mining, inference, and prediction (Vol. 2, pp. 1-758). New York: springer.

Line 346-349 : I am not sure to understand this sentence : why using all data at hand if for example the model selection suggests not using the covariate week. I am maybe missing something here, but this sentence is confusing to me.
Thanks for the comment that allowed us to better clarify the model selection process; please see the changes made at lines 383-393.

Line 355 : what are “means of the final models” : means of which variable ? of the estimated effect of covariate ? across space ?
Here “by means” was intended as a synonym of “through”. The sentence was rephrased, please see lines 402-403.

Line 384-388 : One of the objective of the paper is to “produce standardized CPUEs, to be used as reliable inputs for population dynamic and ecosystem models” (line 149). But does the population dynamic model has a seasonal resolution? if not, I do not think that the authors can say they provided information to feed the population dynamic. Because here the authors provided standardized CPUE for each survey. I think that most of the readers would have expected an annual global index of abundance from this study, and maybe a plot comparing the previous and the new standardized CPUE.
Thanks for the precious comment, please see reply to Major Comments 2 about potential use of seasonal indices; furthermore please note that comparisons between raw and standardized CPUE (by season/survey) have been provided in Figure 8.

Line 600:601: “ in this study GAMs were selected as the best ….” it suggested that the authors have tested other models, did they ?
Also yes GAM are great but other models used for species distribution models can been used to fill the gaps of a time-serie by using autoregressive processes (Thorson, 2019; Anderson et al., 2022).
Thanks a lot for the precious comments that will be also helpful for future development (sentence added in the “Filling the gaps of a time series through standardization with GAMs” section within the Discussion chapter at lines 674-677) and please take in account replies to Major Comments 6


References

Anderson, S. C., Ward, E. J., English, P. A., and Barnett, L. A. K. 2022. sdmTMB: an R package for fast, flexible, and user-friendly generalized linear mixed effects models with spatial and spatiotemporal random fields. preprint. Ecology. <http://biorxiv.org/lookup/doi/10.1101/2022.03.24.485545> (Accessed 29 March 2022).
Brodie, S. J., Thorson, J. T., Carroll, G., Hazen, E. L., Bograd, S., Haltuch, M. A., Holsman, K. K., et al. 2020. Trade-offs in covariate selection for species distribution models: a methodological comparison. Ecography, 43: 11–24.
Potts, S. E., and Rose, K. A. 2018. Evaluation of GLM and GAM for estimating population indices from fishery independent surveys. Fisheries Research, 208: 167–178.
Thorson, J. T. 2019. Guidance for decisions using the Vector Autoregressive Spatio-Temporal (VAST) package in stock, ecosystem, habitat and climate assessments. Fisheries Research, 210: 143–161.
