# Supplementary material for: Accounting for environmental and fishery management factors when standardizing CPUE data from a scientific survey: A case study for Nephrops norvegicus in the Pomo Pits area (Central Adriatic Sea)
Source: PLoS One. 2022 Jul 14;17(7):e0270703. doi: 10.1371/journal.pone.0270703 (PMC9282463; doi:10.1371/journal.pone.0270703)
Supplement: Attachment — Submitted filename: Chiarini et al. (PlosONE)_Response to Reviewers.docx [file pone.0270703.s004.docx]

**Review Comments to the Author:**

Reviewer #2:  In this study the authors applied GAMs to produce specific standardized indices, in number and weight, accounting for the effects of both environmental variables and fishery management actions. I believe that this study can be very interesting due to the need to standardize survey data, an increasing necessity mainly in the pandemic period where a lot of surveys have often been made but in a wrong period.
The authors have done a very comprehensive job of revising their manuscript and have addressed all previous concerns from me and the other referees. Although I enjoyed the previous version as well, the current version shows some more improvements making it better matching the required journal standards.
For this reason, I recommend acceptance for this manuscript as it is.

Reviewer #3: The study by Chiarini et al., presents an analysis to standardize N. norvegicus CPUE from 2 surveys from 2012 to 2019 using GAM models. In particular the authors i) investigate how the environmental variables and the different management strategies can influence abundance and distribution of Nephrops, ii) produce standardized CPUEs, to be used as reliable inputs for population dynamic and ecosystem models. The data are coming from two seasonal surveys, the areas associated with each survey being under different management measures.
I found the objectives of the study interesting. The questions addressed in this manuscript seem to be very important for the ecosystem studied (Pomo Pits area (Central Adriatic Sea)).
I appreciated that the authors answered and accounted for all my comments, that is why I recommend minor revision. So with the exception of some comments/questions described below, I found the manuscript’s study design robust and its conclusions fair. Below I sum up my main remaining comments that I would like the authors to answers.

Line 268-269 : 2.6 hauls removed per year : Which % does it represent per year because I guess it is represent a different % between 2012 (17 hauls) and 2013 (10 hauls)

Thanks for the comment; the numbers of removed hauls and percentages for each year have been now added in the text.

Line 430-437 : I am wondering : If the objective is to provide indices to feed stock assessment model, instead of averaging across the predicting grid, is it not more appropriate to multiplicate each density by the area of the grid and then sum it across the grid to have a the total abundance (biomass) of the area

Actually, the GAM models produce CPUEs estimates and within this paper we also wanted to show a comparison between the newly obtained CPUEs and the raw ones (see Fig. 8); to do so, since the surface of each cell is equal (we tried to make it more clear in the text, please see line 438), averaging CPUEs per cell or calculate the total abundance and then divide by the total surface of the study area would lead to the same results in terms of numbers. In the same way, the total abundance to be used for stock assessment purposes would be easily obtained by multiplying the average CPUE for the total surface of the study area.

Line 444-447 : I am not familiar with an ANOVA test of the AIC values. Is there any paper justifying this approach? To my knowledge if the difference between AIC is 5, the model with the lowest AIC is considered better.

Thanks for the comment. It is well known that models with lower AIC must be preferred to the models with higher AIC (Weakliem, 2016). In our case, since we ran each model 100 times, we obtained four groups (four models) of 100 AIC values each to be compared. To detect significant differences among groups, we ran ANOVA with post hoc tests taking the AIC values as the dependent variable and the models as levels of the factor. We are not aware of any paper making this specific analysis (AIC versus models), however ANOVA is a classic technique to compare groups of data.

Weakliem DL. Hypothesis Testing and Model Selection in the Social Sciences. 2016

Line 643-649 : I will move this information, in the paragraph “purpose of the study”. Because I think the reader does not understand why he goes through all those repeated analysis for both abundance and biomass. And results seem very similar for abundance and biomass ( I feel that it is only on line 618, we really identify there is a difference). In the discussion paragraph, I will rather say if there is discrepancy or not (and sum up what are the main differences), instead of saying why the authors carried out standardization on both abundance and biomass (the “why” should be in Introduction or Method)

Again thanks for valuable comment: as suggested we moved (and adapted) this paragraph in the Materials and Methods section, we added a sentence in the results (please see lines 641-644) and tried to better discuss it (at new lines 650-656).

Line 828 : It is more a comment than a request. I am curious to have the authors point of view. Because depending on the years, the sampling (number of hauls) is not homogeneous (in year 2012, there are 17 hauls whereas in 2013 there is a 10 hauls) can we think that the year effect in the model is confounded with a sampling effect ?

Thanks for the interesting question! From our point of view, in this case there is not a confounding effect between year and sampling according to the experimental design; indeed, the ratio between the number of hauls in the different geographical areas was maintained almost constant and there is no reason to believe that the mean CPUE is biased by the number of hauls.
